# Supplementary material for: 1-(1-Arylethylpiperidin-4-yl)thymine Analogs as Antimycobacterial TMPK Inhibitors
Source: Molecules. 2020 Jun 17;25(12):2805. doi: 10.3390/molecules25122805 (PMC7356956; doi:10.3390/molecules25122805)
Supplement: Supplementary file 1 [file molecules-25-02805-s001.pdf]

molecules

MDPI

Article

1-(1-Arylethylpiperidin-4-yl)thymine Analogs as Antimycobacterial TMPK Inhibitors

Yanlin Jian [1], Fabian Hulpia [1], Martijn D. P. Risseeuw [1], He Eun Forbes [2], Guy Caljon [3], Hélène Munier-Lehmann [4], Helena I. M. Boshoff [2] and Serge Van Calenbergh [1,*]

[1] Laboratory for Medicinal Chemistry (FFW), Ghent University, Ottergemsesteenweg 460, B-9000 Gent, Belgium; yanlin.jian@ugent.be (Y.J.); fabian.hulpia@ugent.be (F.H.); martijn.risseeuw@ugent.be (M.D.P.R.)

[2] Tuberculosis Research Section, Laboratory of Clinical Immunology and Microbiology, National Institute of Allergy and Infectious Disease, National Institutes of Health, 9000 Rockville Pike, Bethesda, MD 20892, United States; grace.chun@nih.gov (H.E.F.); hboshoff@niaid.nih.gov (H.I.M.B.)

[3] Laboratory of Microbiology, Parasitology and Hygiene, University of Antwerp, Universiteitsplein 1 (S7), B-2610 Wilrijk, Belgium; Guy.Caljon@uantwerpen.be

[4] Unit of Chemistry and Biocatalysis, Department of Structural Biology and Chemistry, Institut Pasteur, CNRS UMR3523, 28 Rue du Dr. Roux, CEDEX 15 75724 Paris, France; helene.munier-lehmann@pasteur.fr

* Correspondence: serge.vancalenbergh@ugent.be; Tel.: +32 9 264 81 24

Academic Editor: Athina Geronikaki

Received: 6 May 2020; Accepted: 14 June 2020; Published: 17 June 2020

Table of contents:

Figure S1–S43– NMR spectra of final compounds 2-44

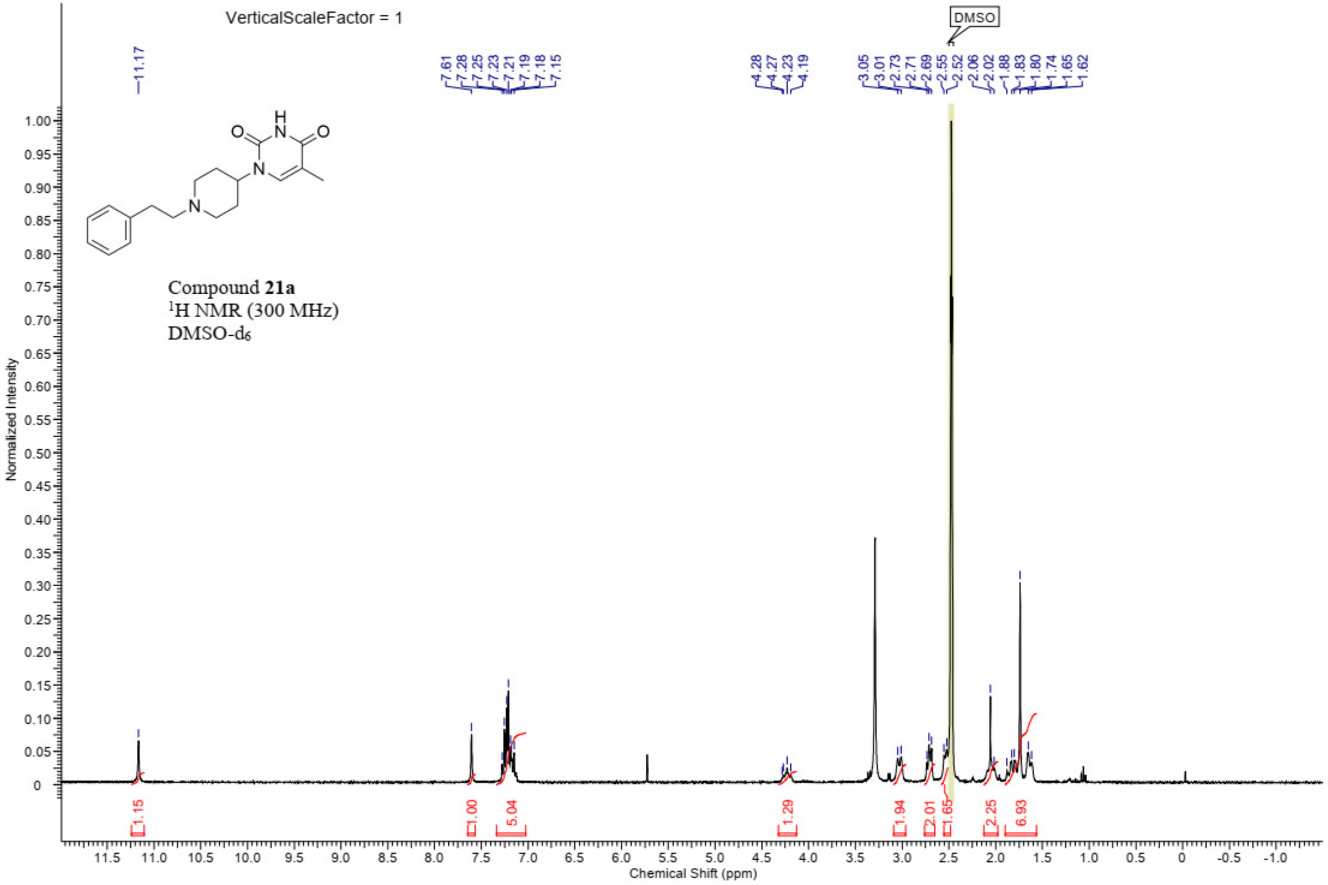

Figure S1. ^{1}H NMR spectrum of compound **21a**.

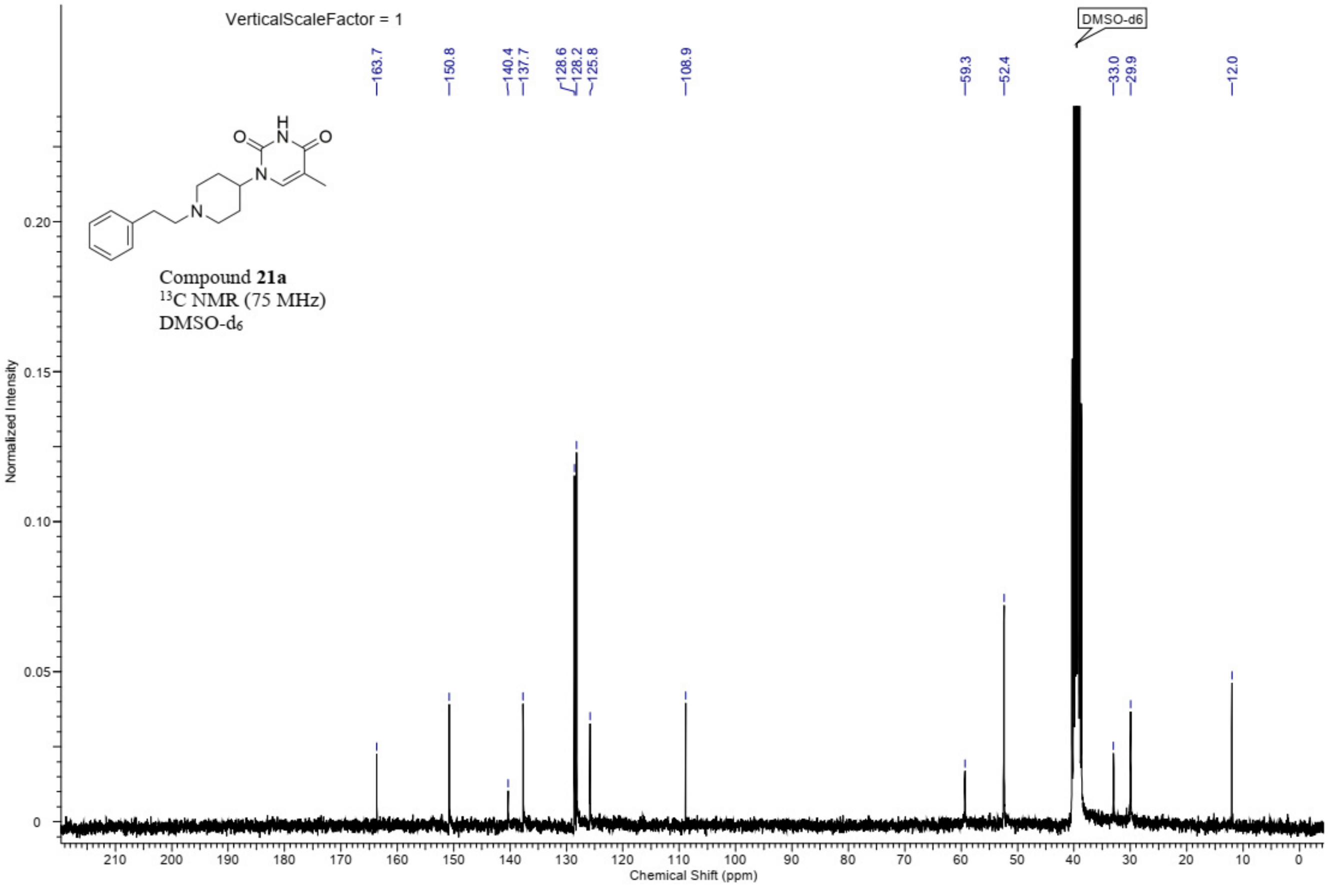

Figure S2. ^{13}C NMR spectrum of compound **21a**.

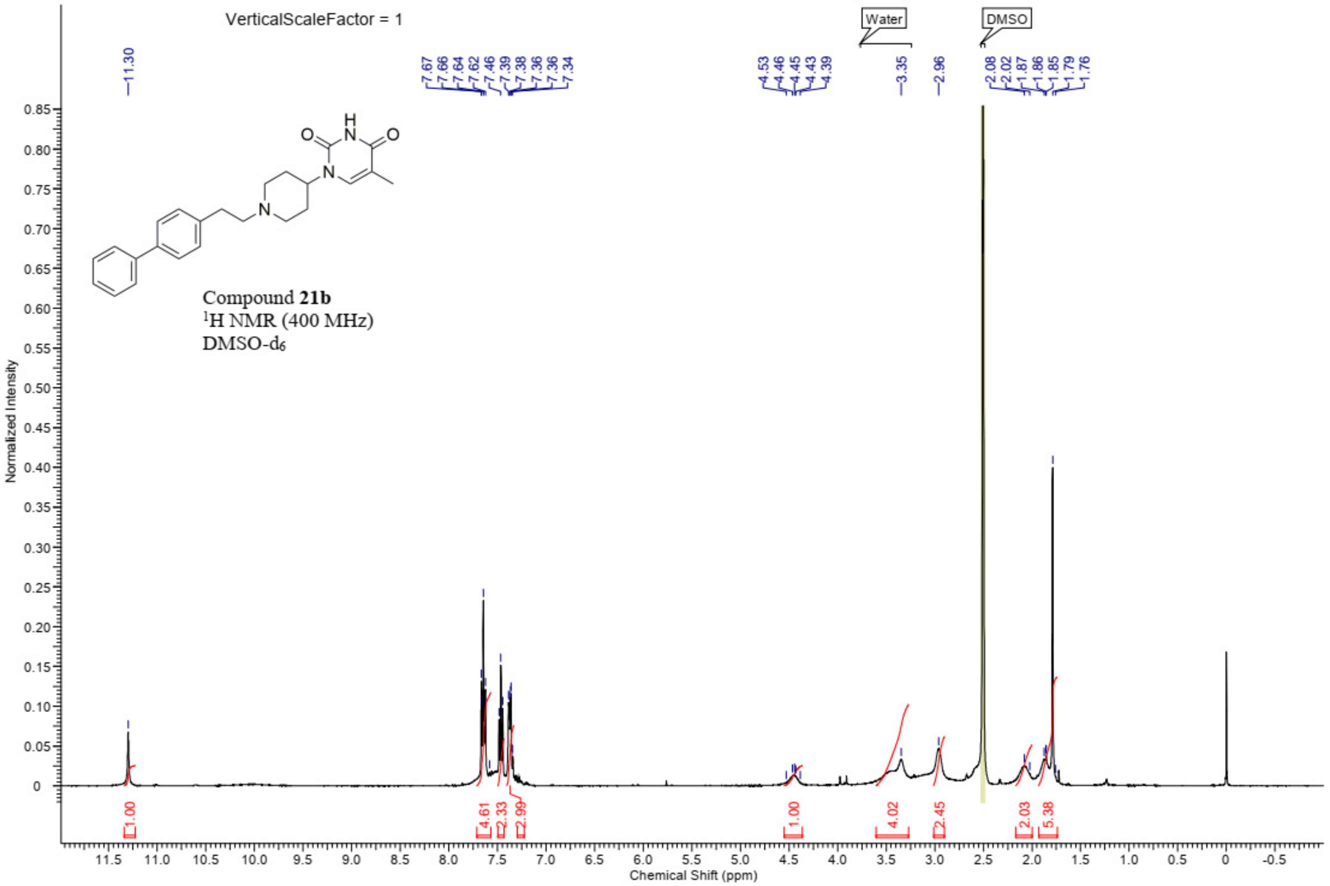

Figure S3. ^{1}H NMR spectrum of compound **21b**.

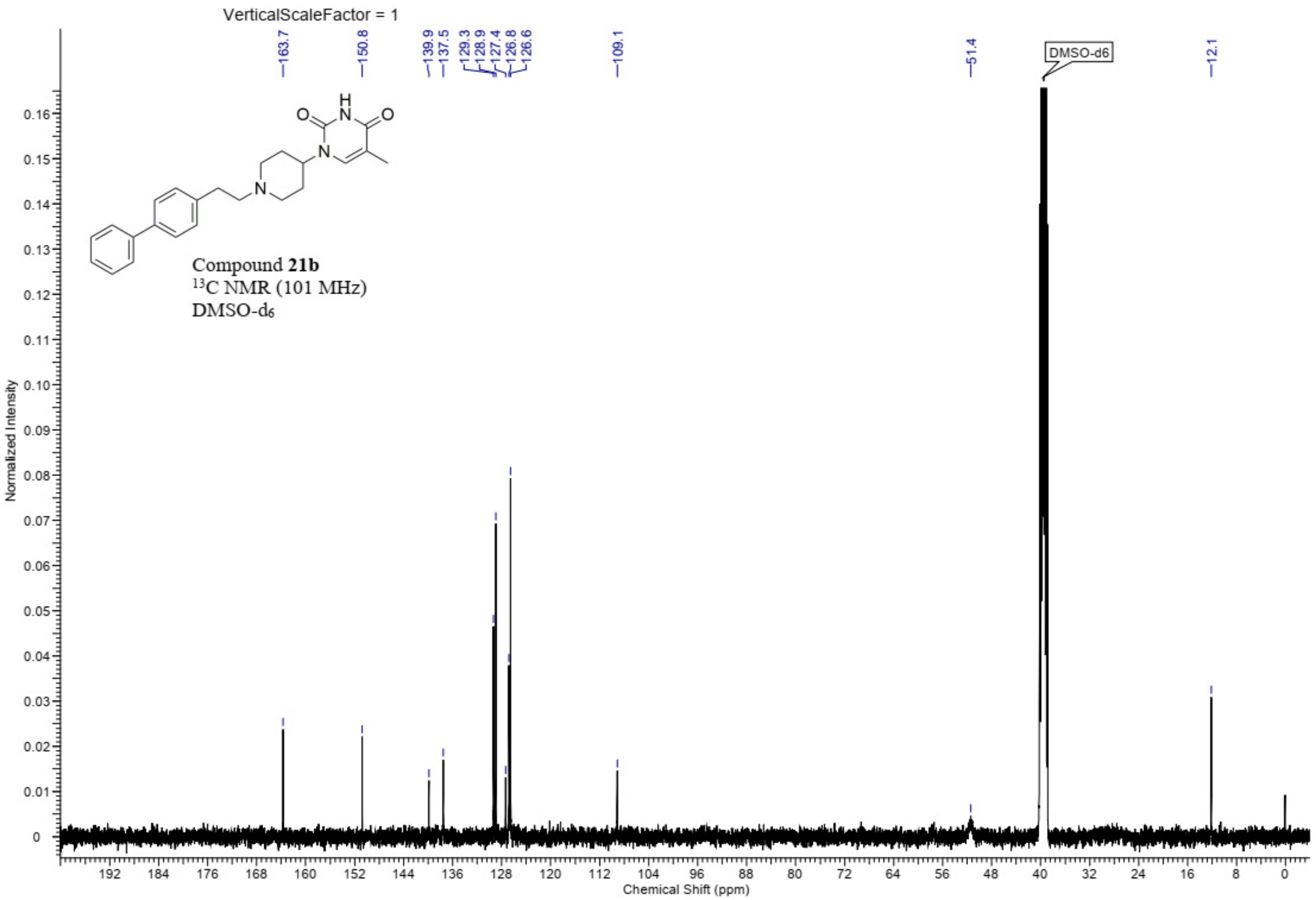

Figure S4. ^{13}C NMR spectrum of compound **21b**.

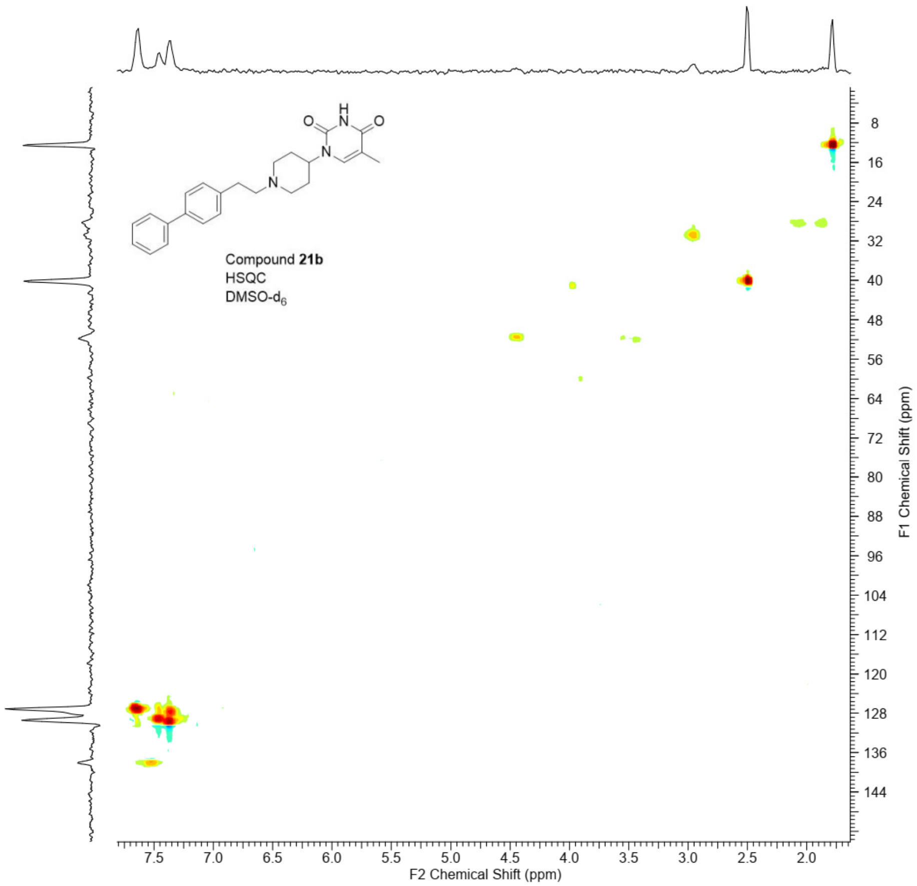

Figure S5. HSQC spectrum of compound **21b**.

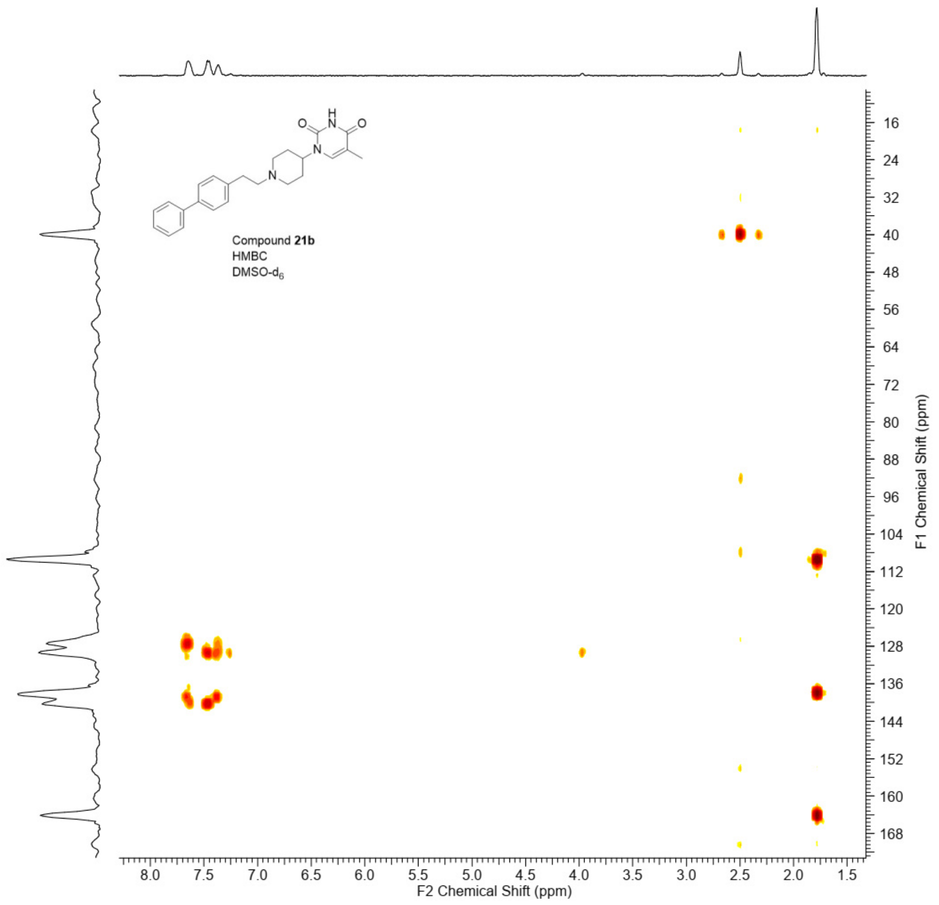

Figure S6. HMBC spectrum of compound **21b**.

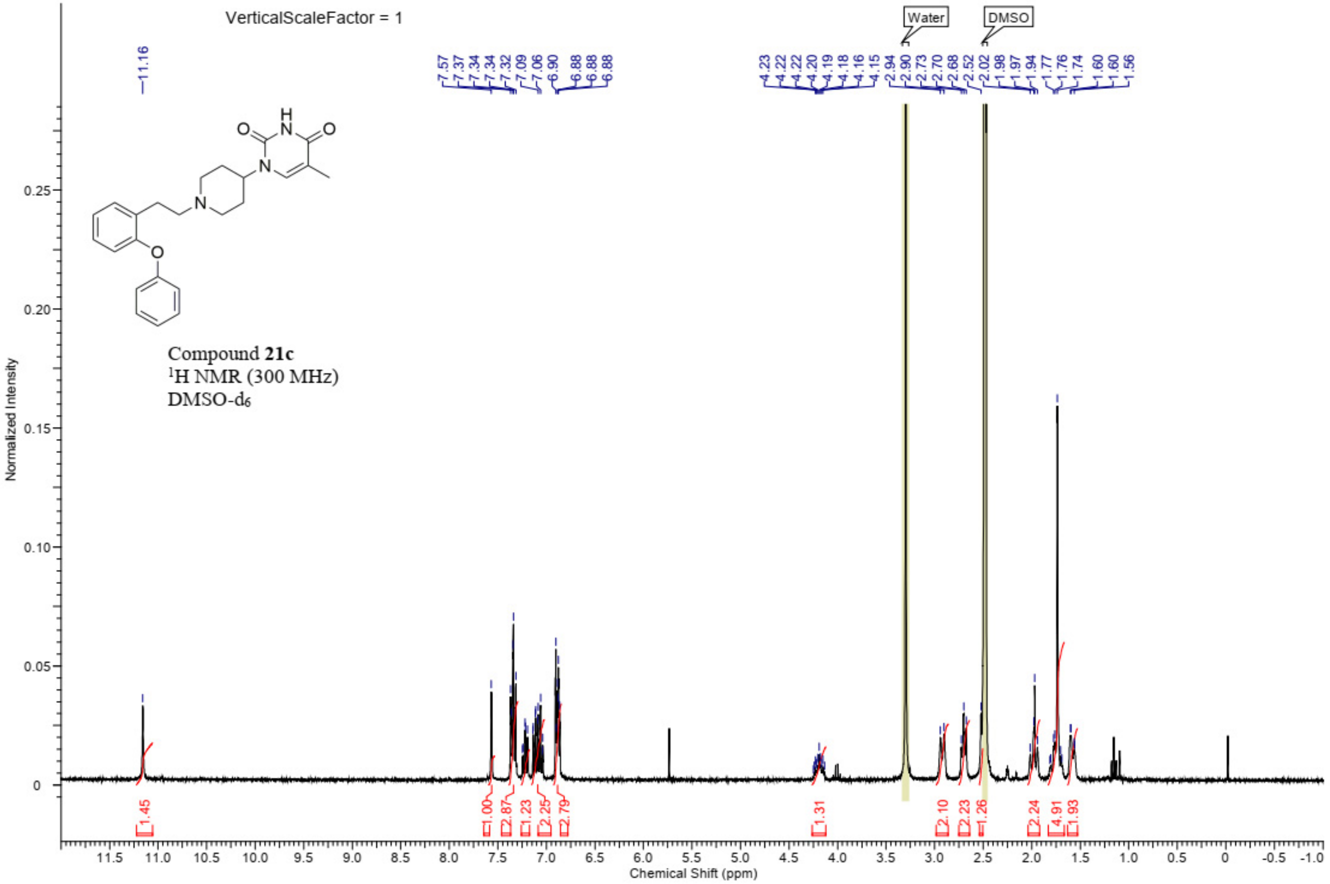

Figure S7. ^{1}H NMR spectrum of compound **21c**.

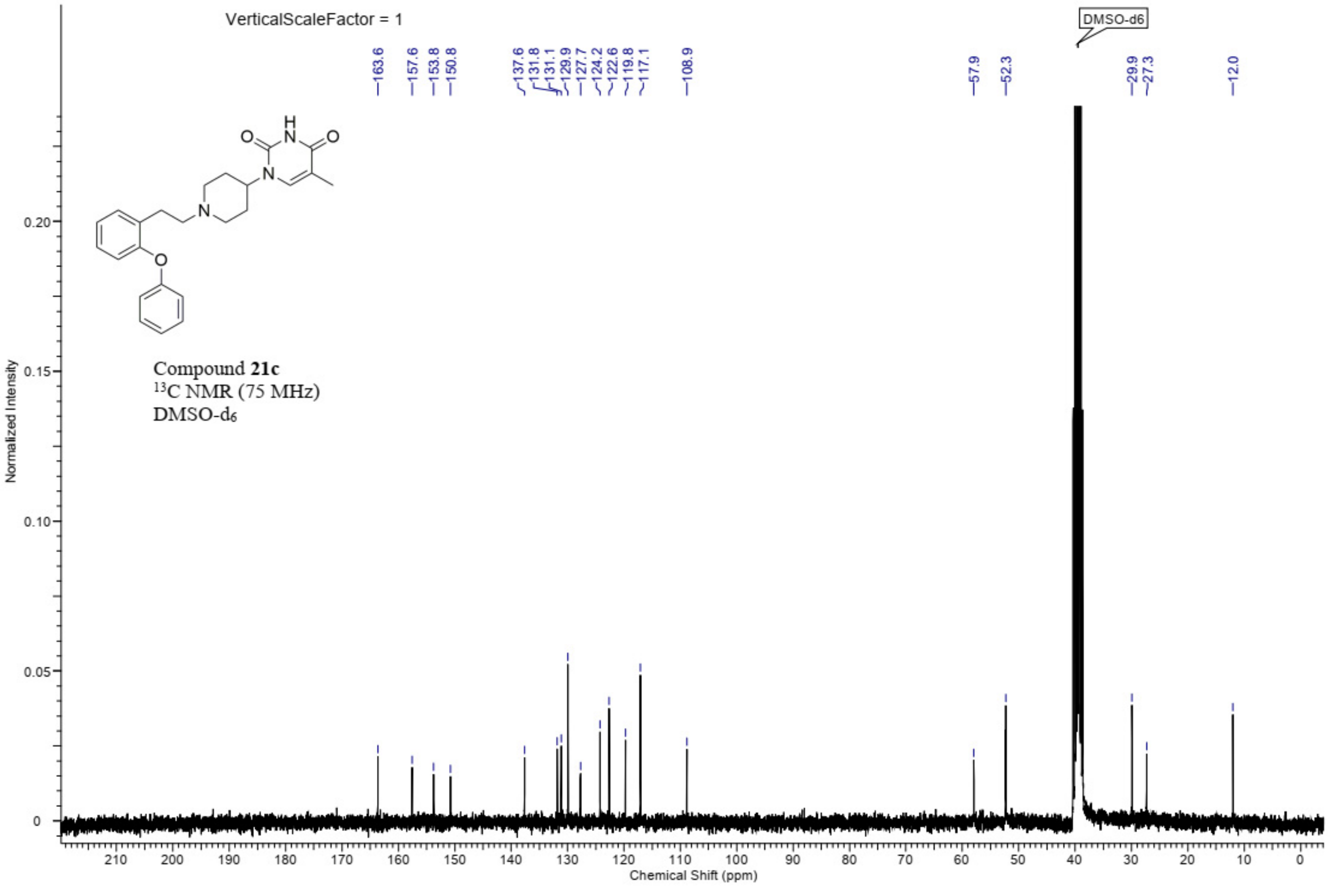

Figure S8. ^{13}C NMR spectrum of compound **21c**.

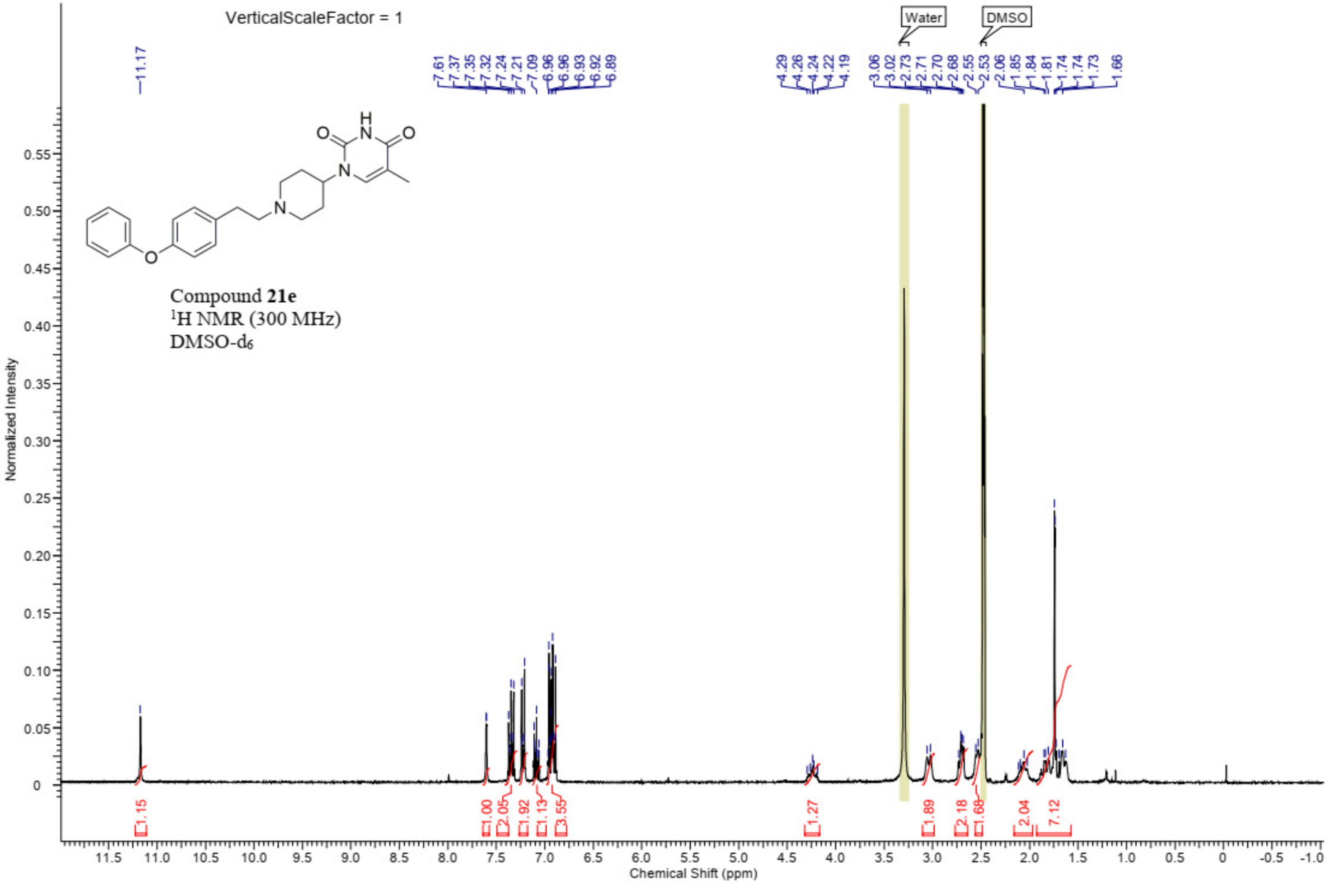

Figure S9. ^{1}H NMR spectrum of compound **21e**.

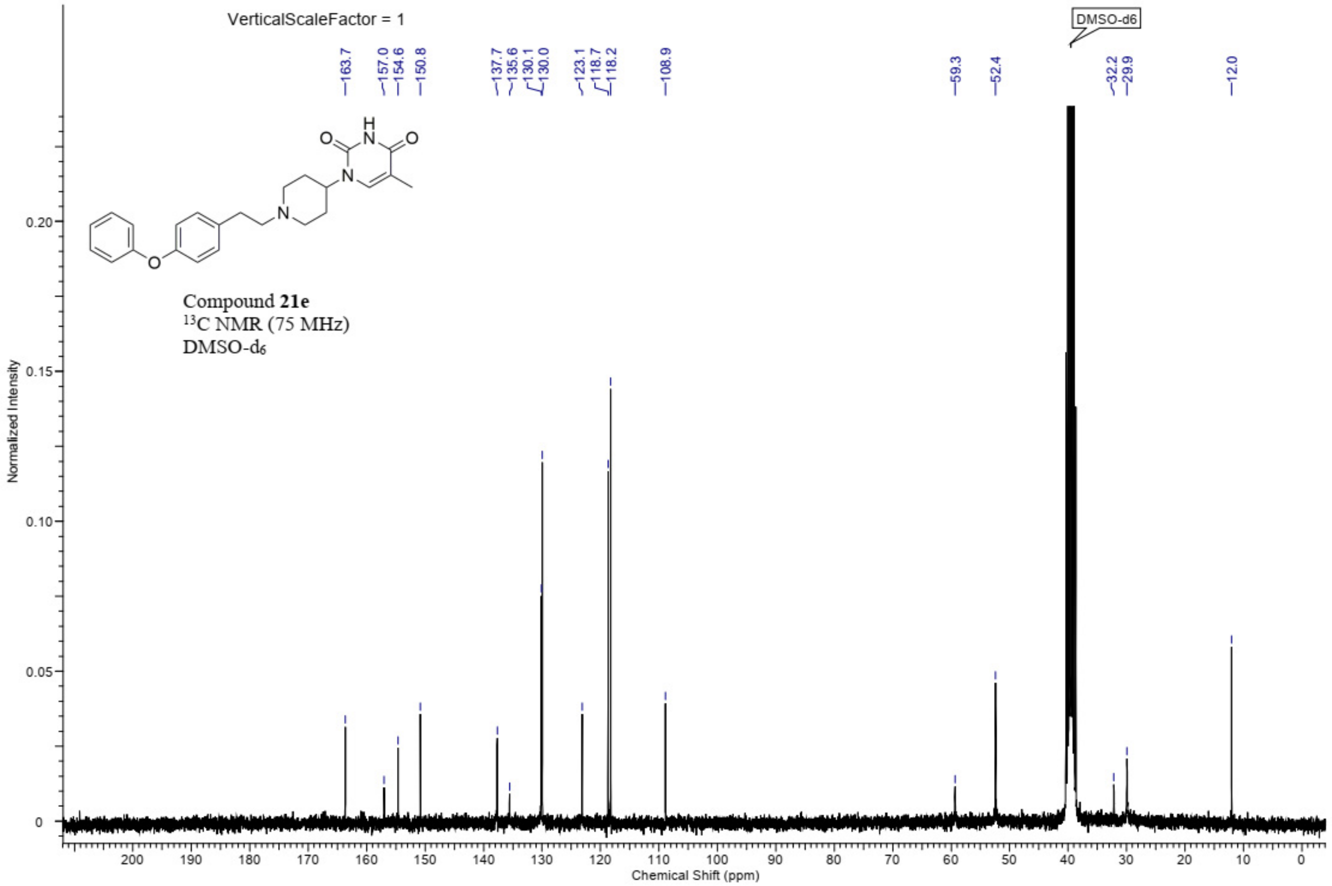

Figure S10. ^{13}C NMR spectrum of compound **21e**.

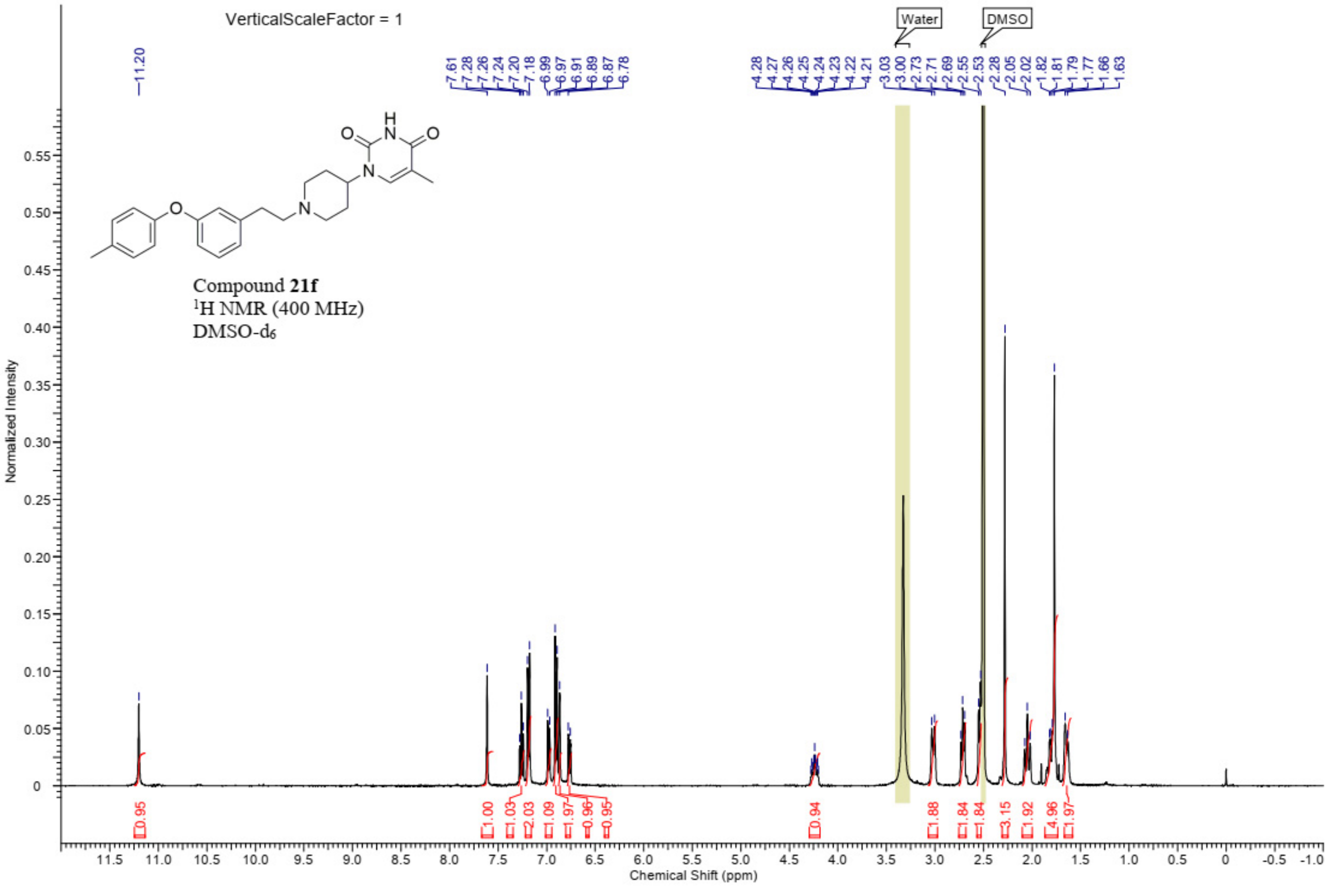

Figure S11. ^{1}H NMR spectrum of compound **21f**.

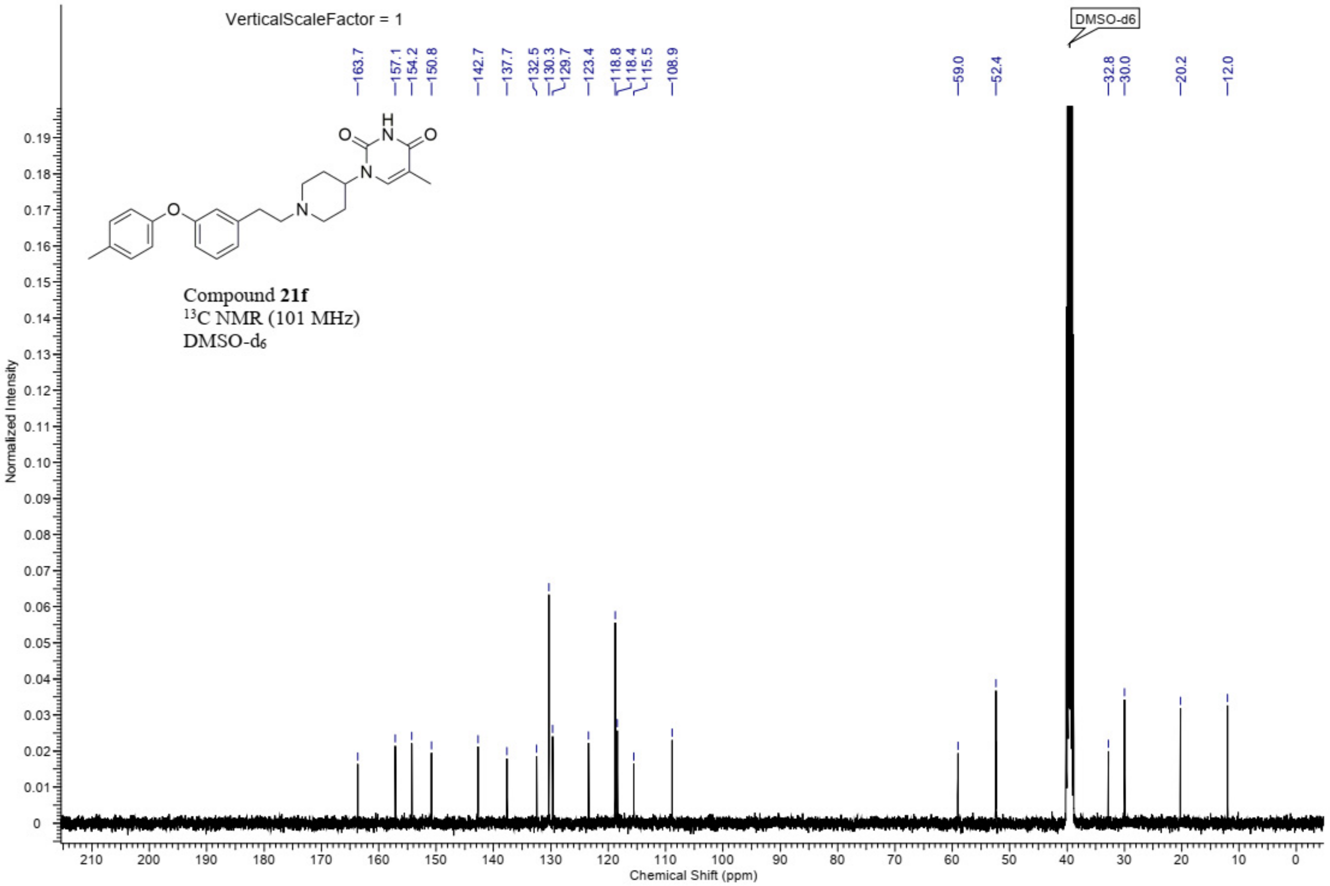

Figure S12. ^{13}C NMR spectrum of compound **21f**.

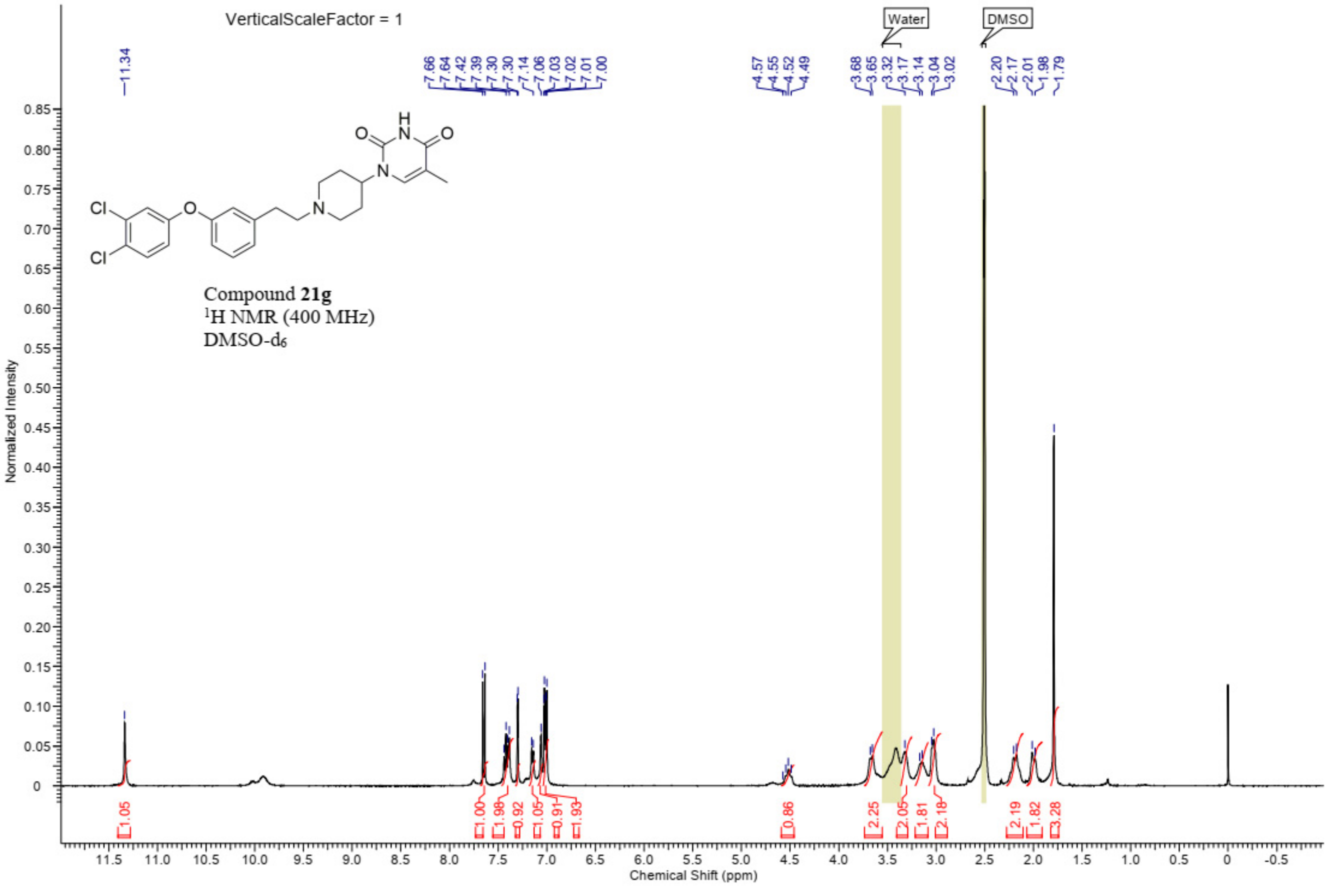

Figure S13. ^{1}H NMR spectrum of compound **21g**.

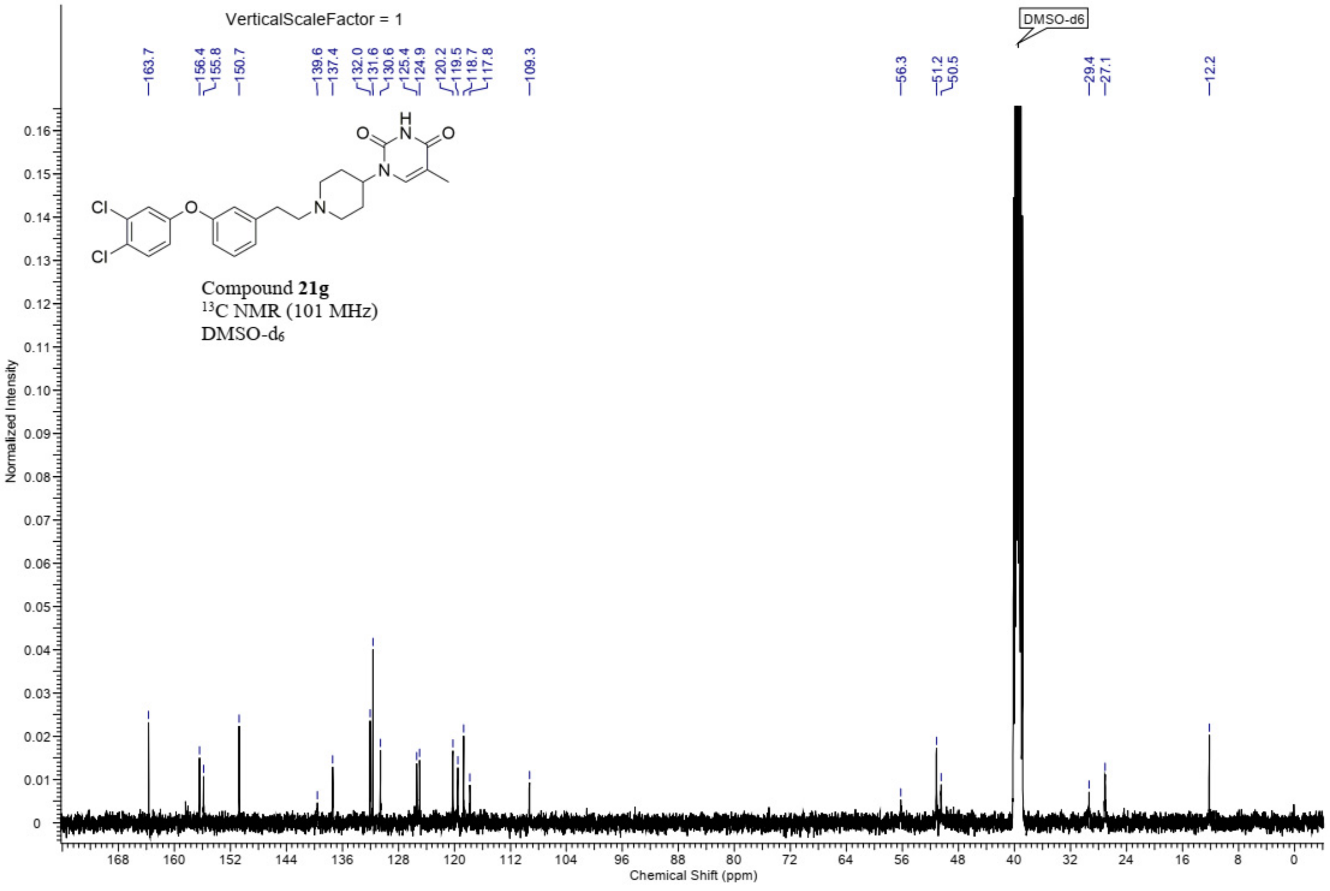

Figure S14. ^{13}C NMR spectrum of compound **21g**.

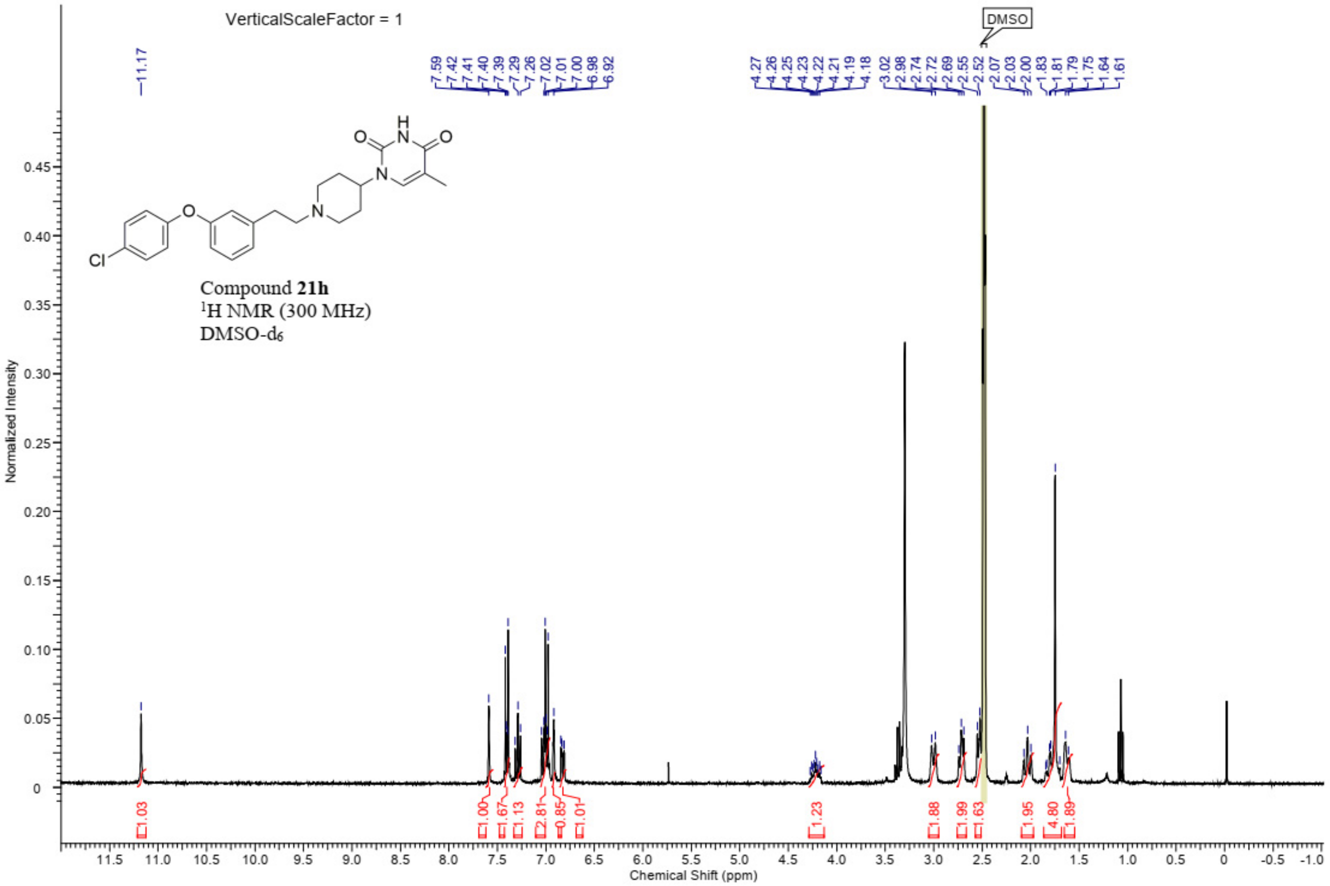

Figure S15. ^{1}H NMR spectrum of compound **21h**.

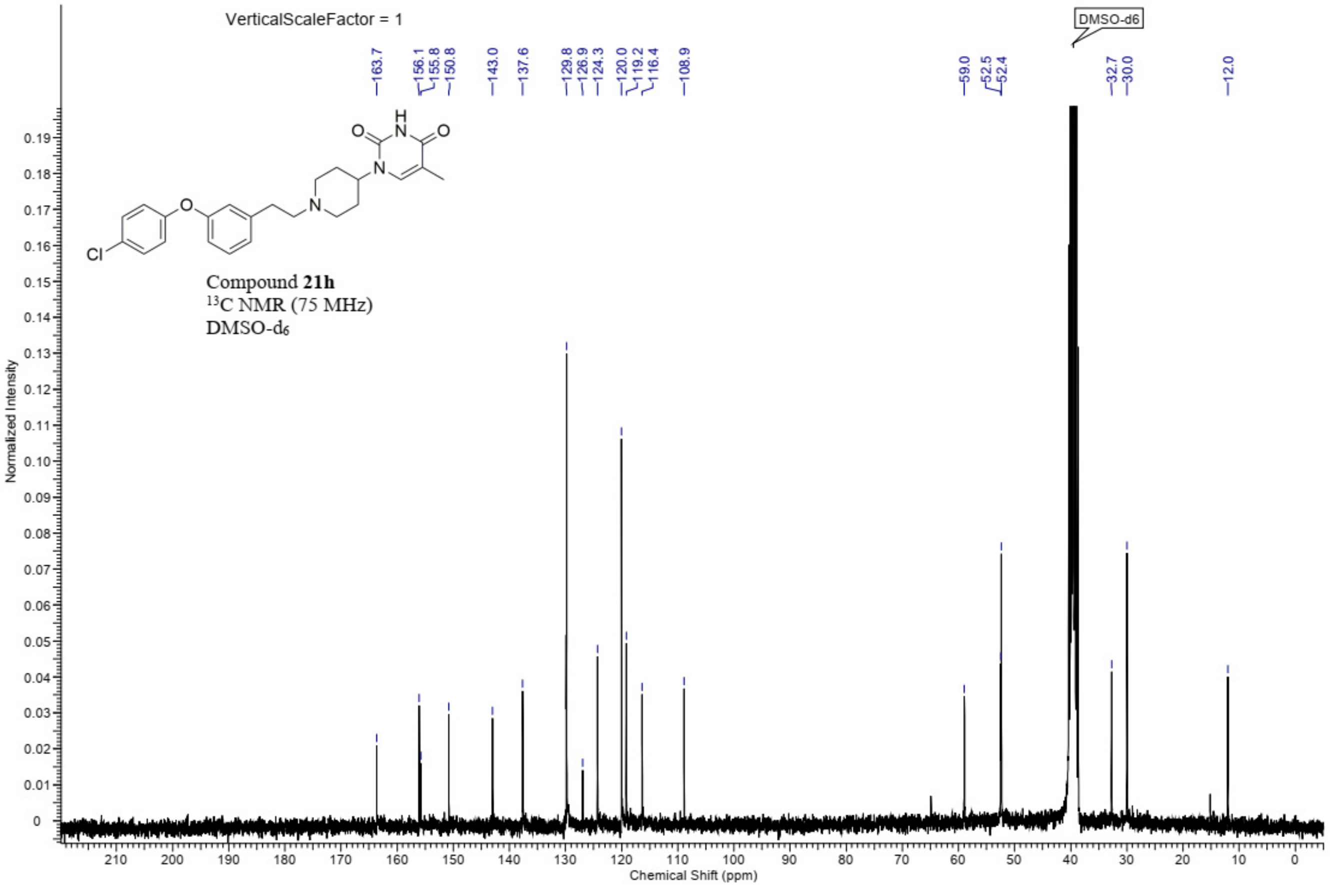

Figure S16. ^{13}C NMR spectrum of compound **21h**.

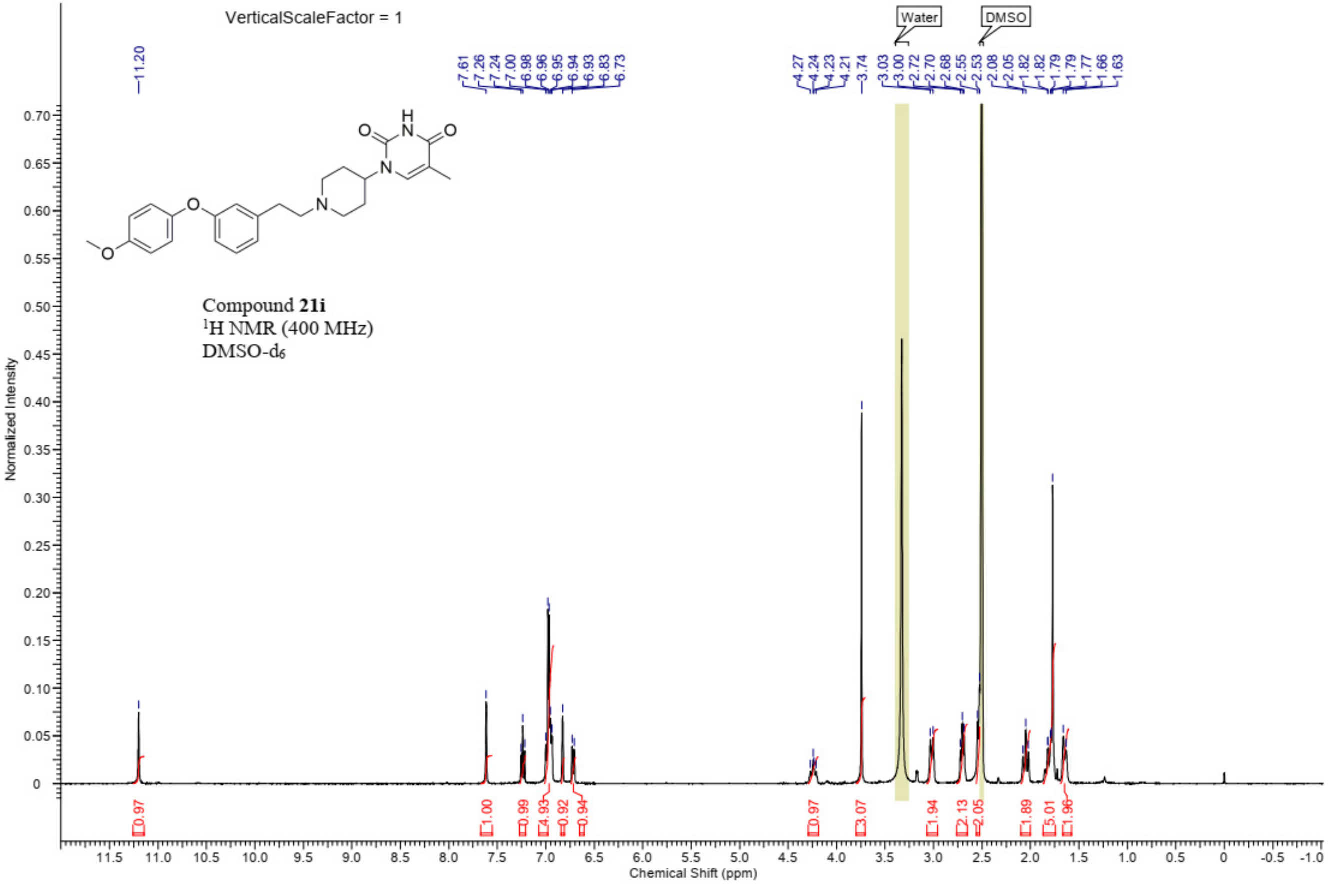

Figure S17. ^{1}H NMR spectrum of compound **21i**.

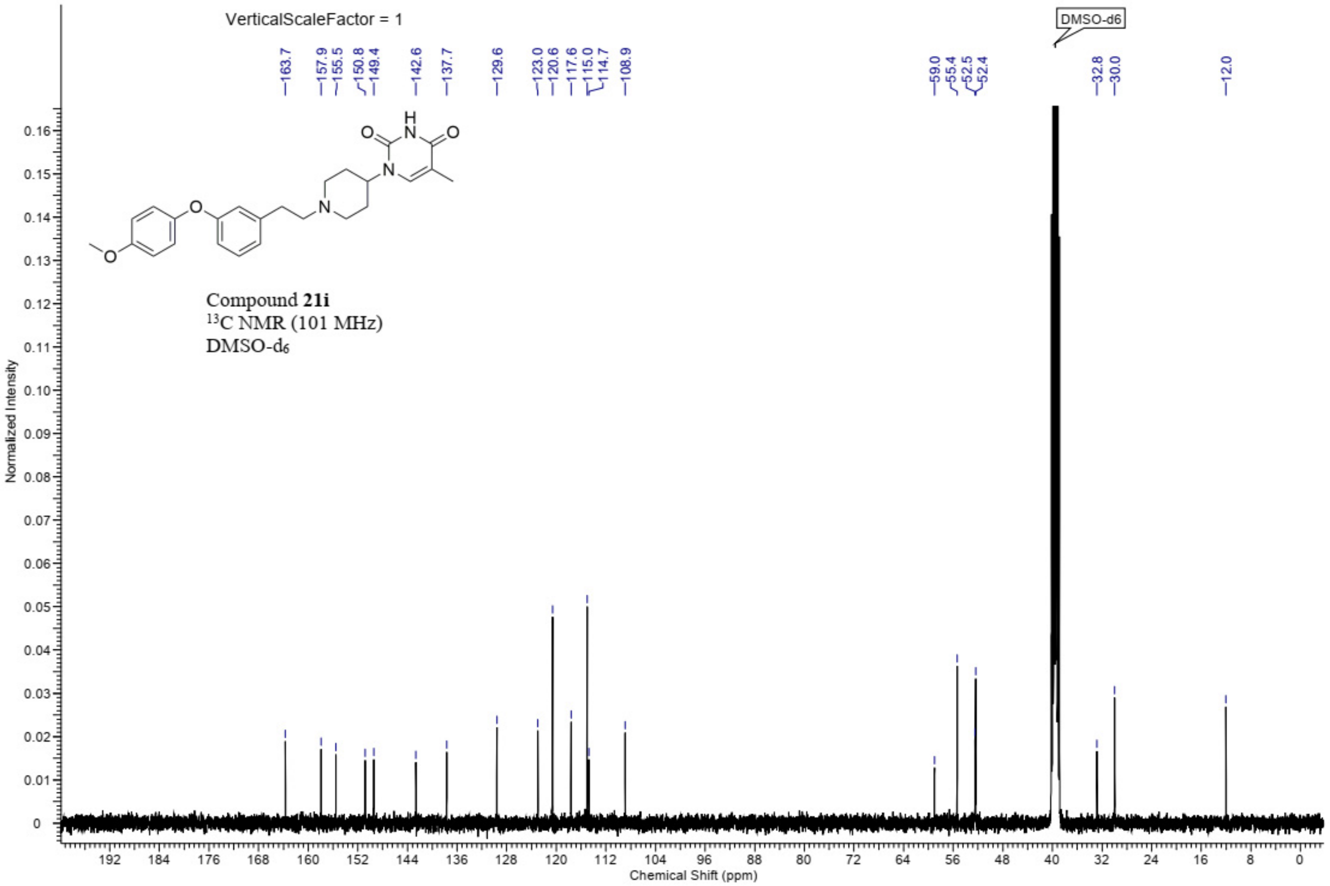

Figure S18. ^{13}C NMR spectrum of compound **21i**.

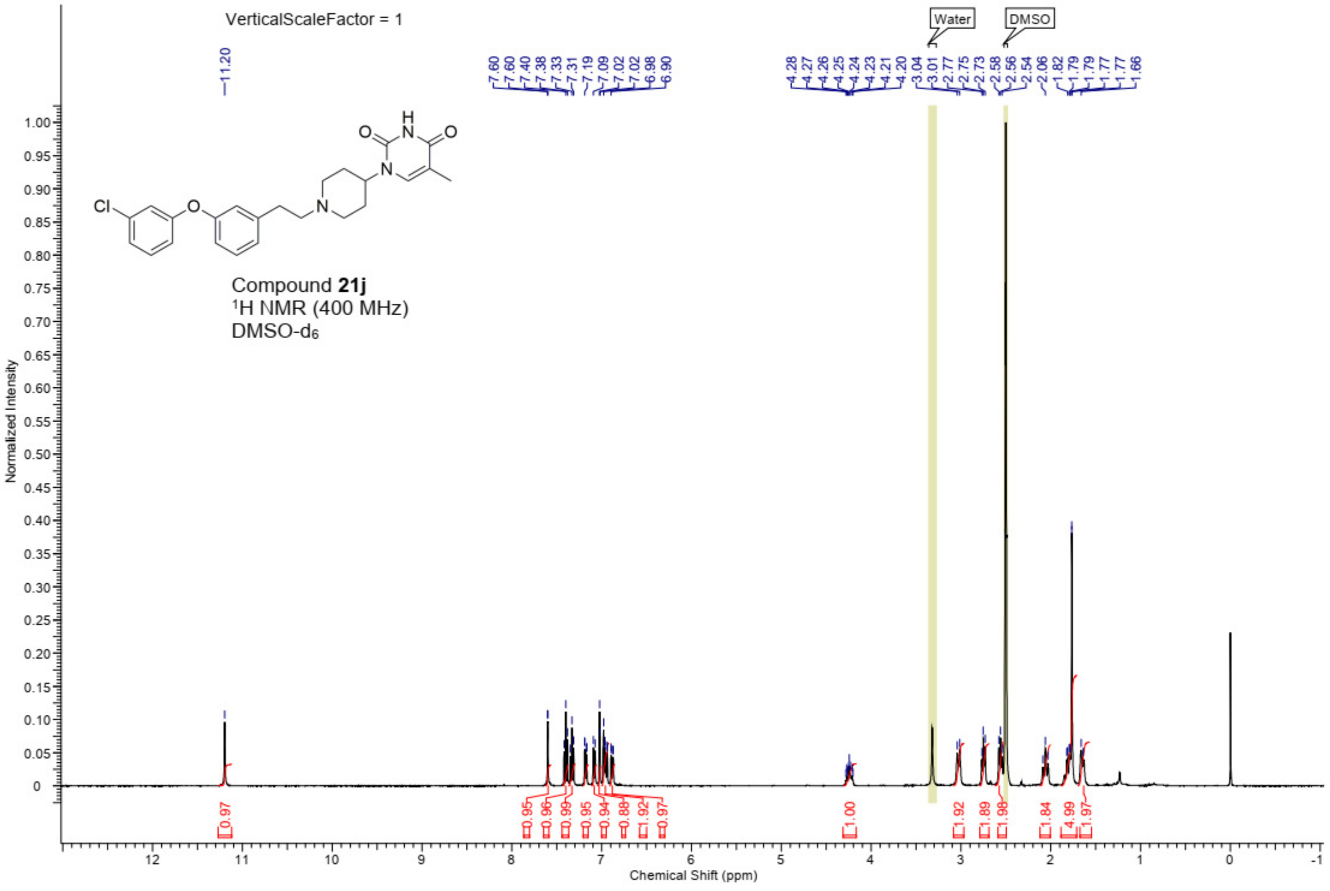

Figure S19. ^{1}H NMR spectrum of compound **21j**.

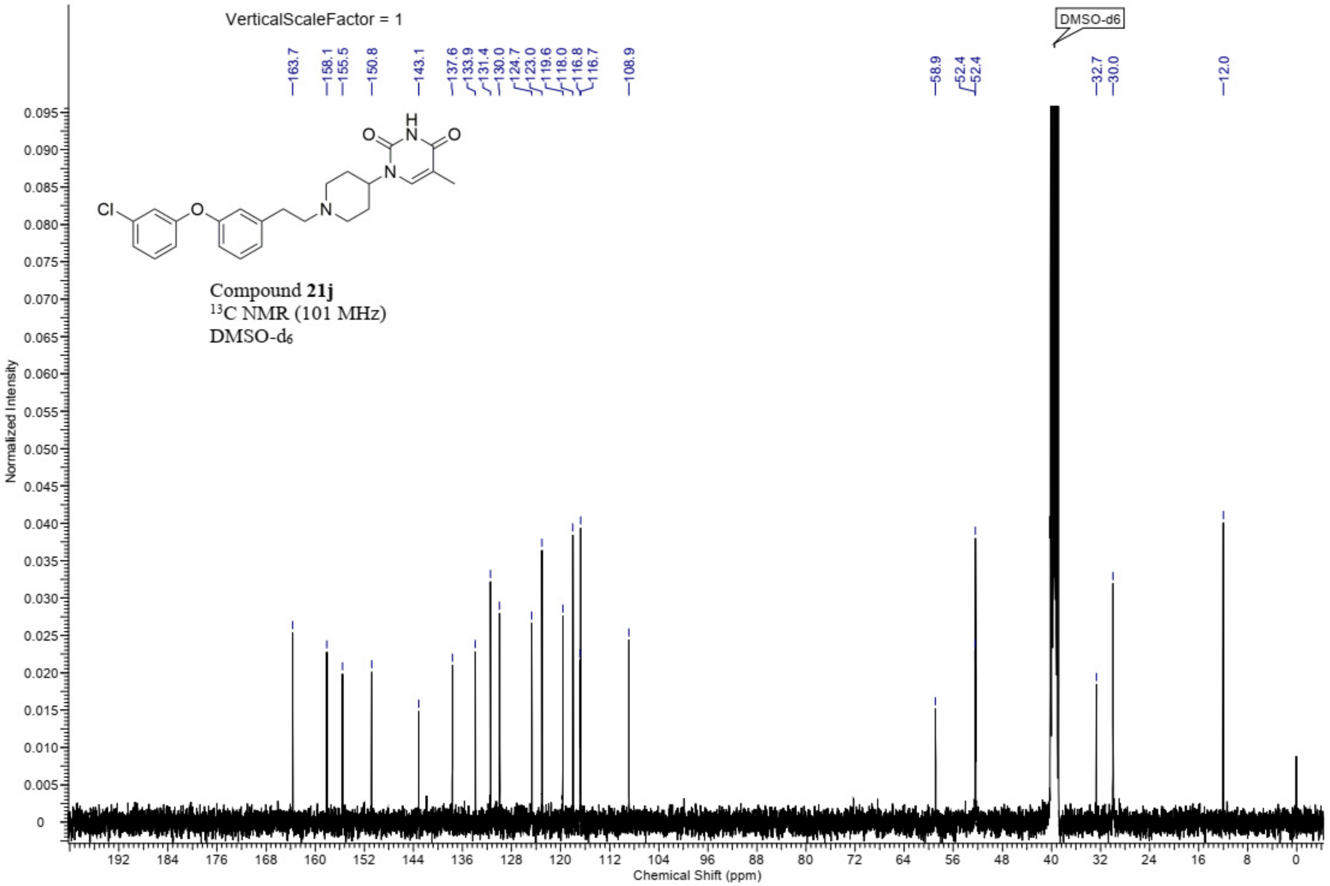

Figure S20. ^{13}C NMR spectrum of compound **21j**.

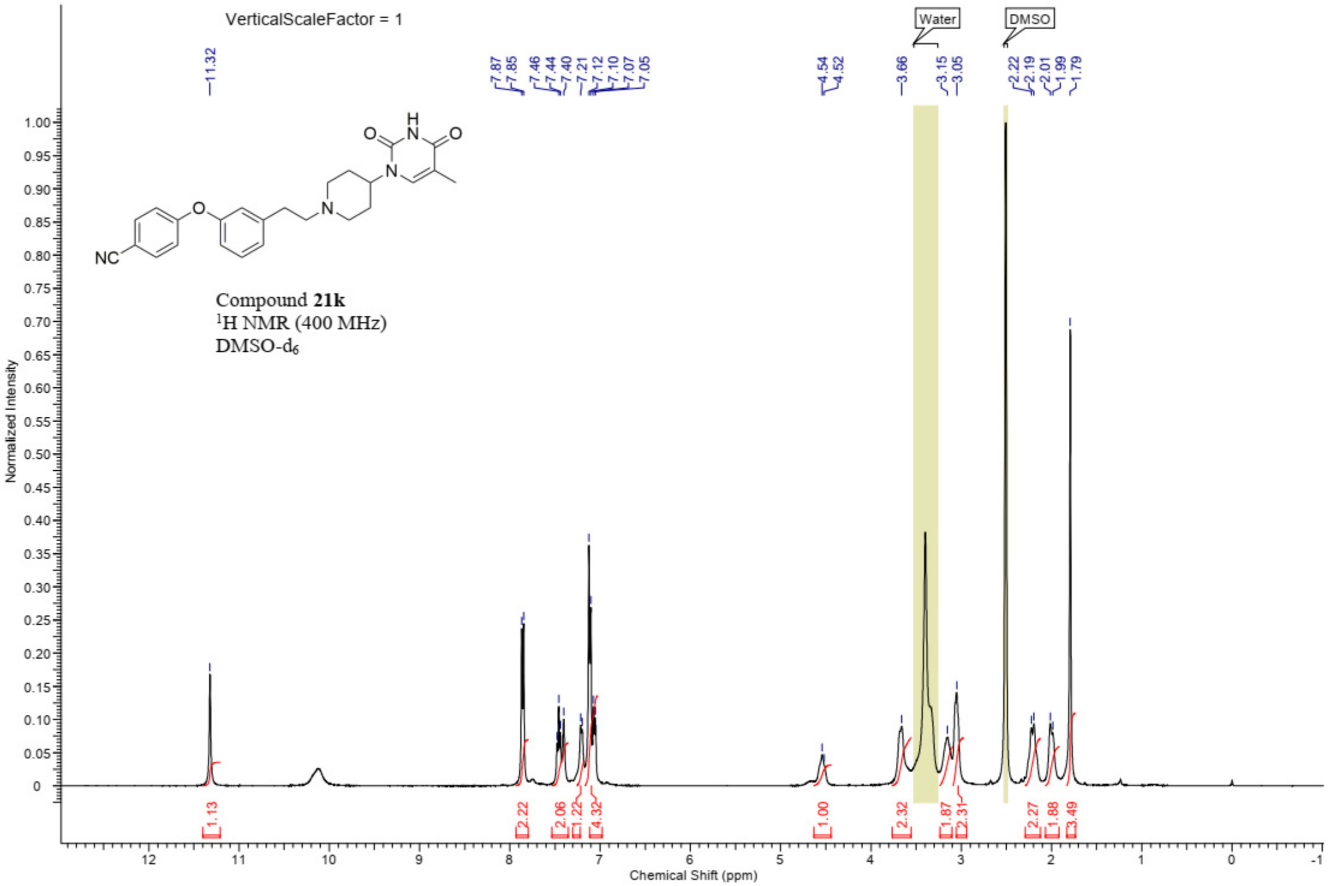

Figure S21. ^{1}H NMR spectrum of compound **21k**.

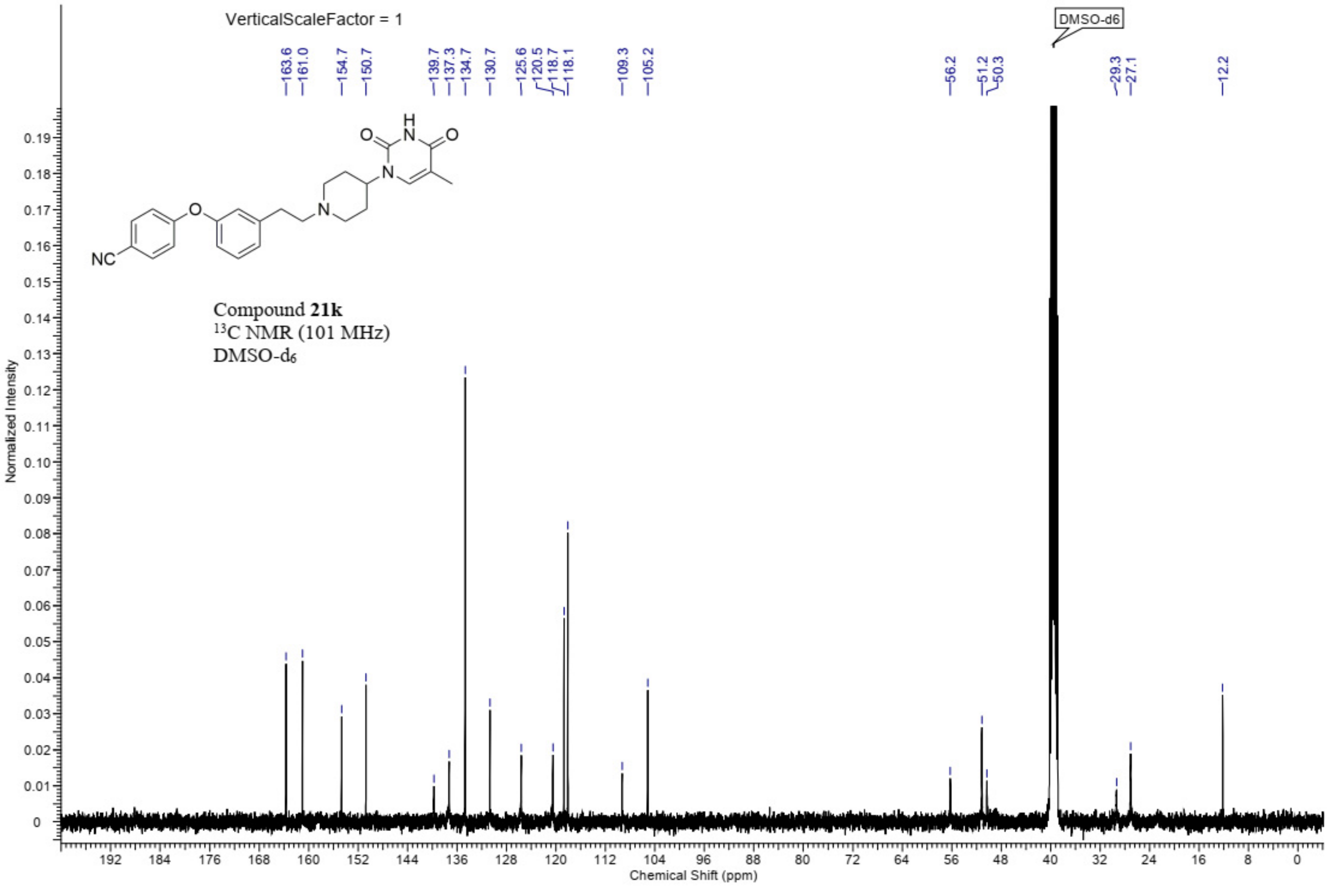

Figure S22. ^{13}C NMR spectrum of compound **21k**.

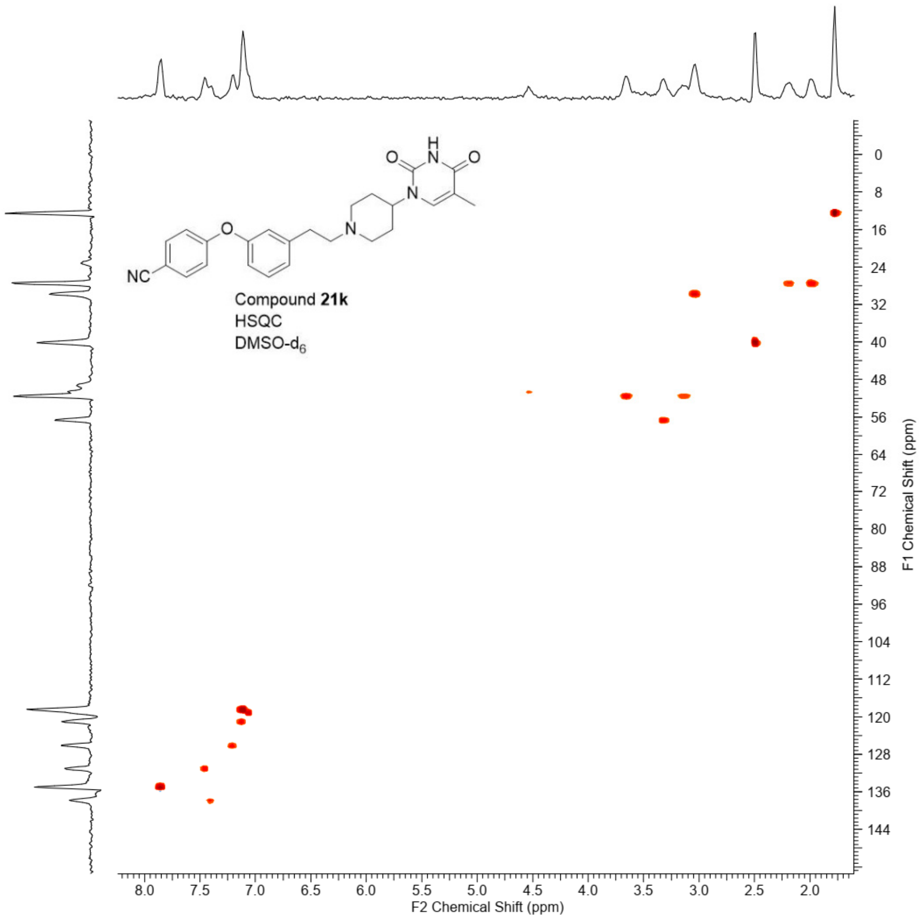

Figure S23. HSQC spectrum of compound **21k**.

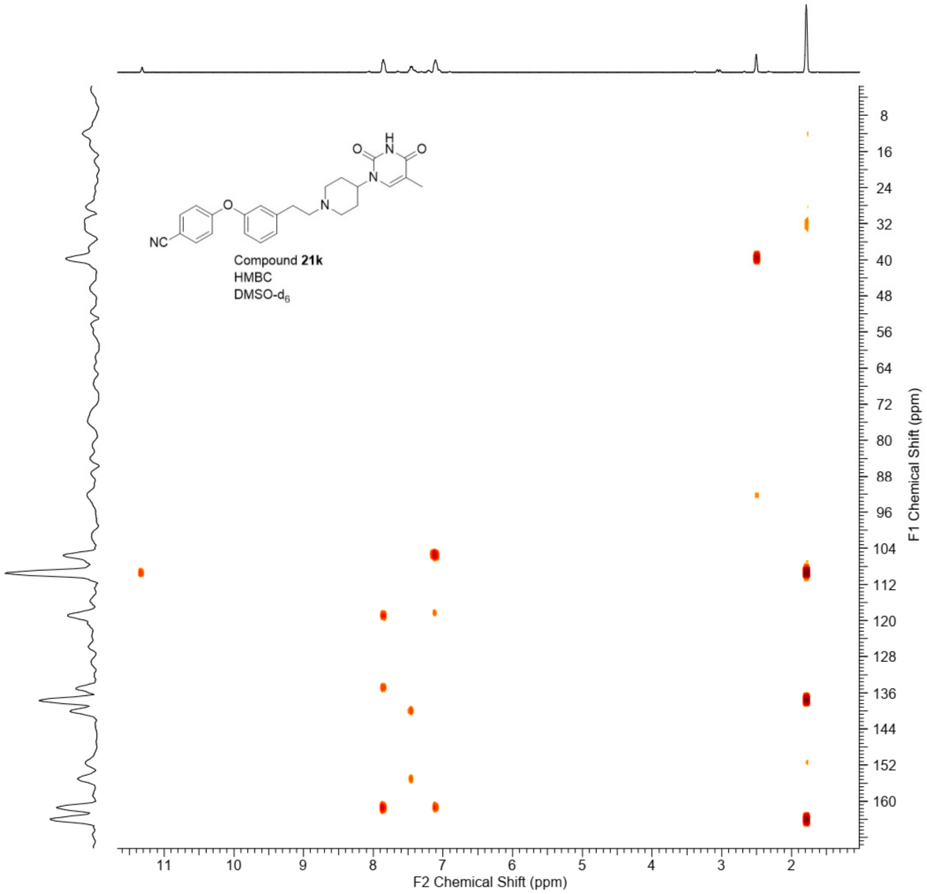

Figure S24. HMBC spectrum of compound **21k**.

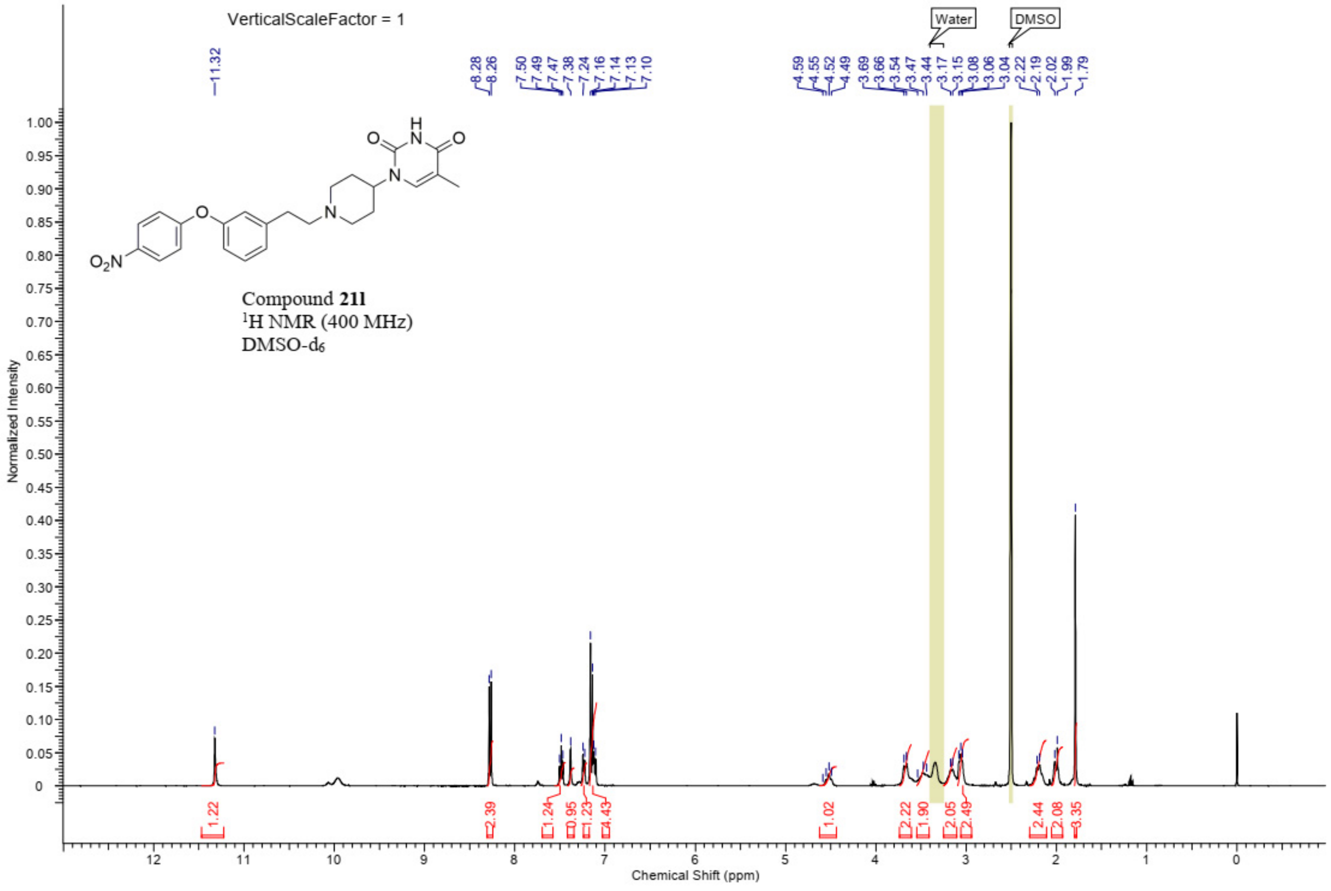

Figure S25. ^{1}H NMR spectrum of compound **21l**.

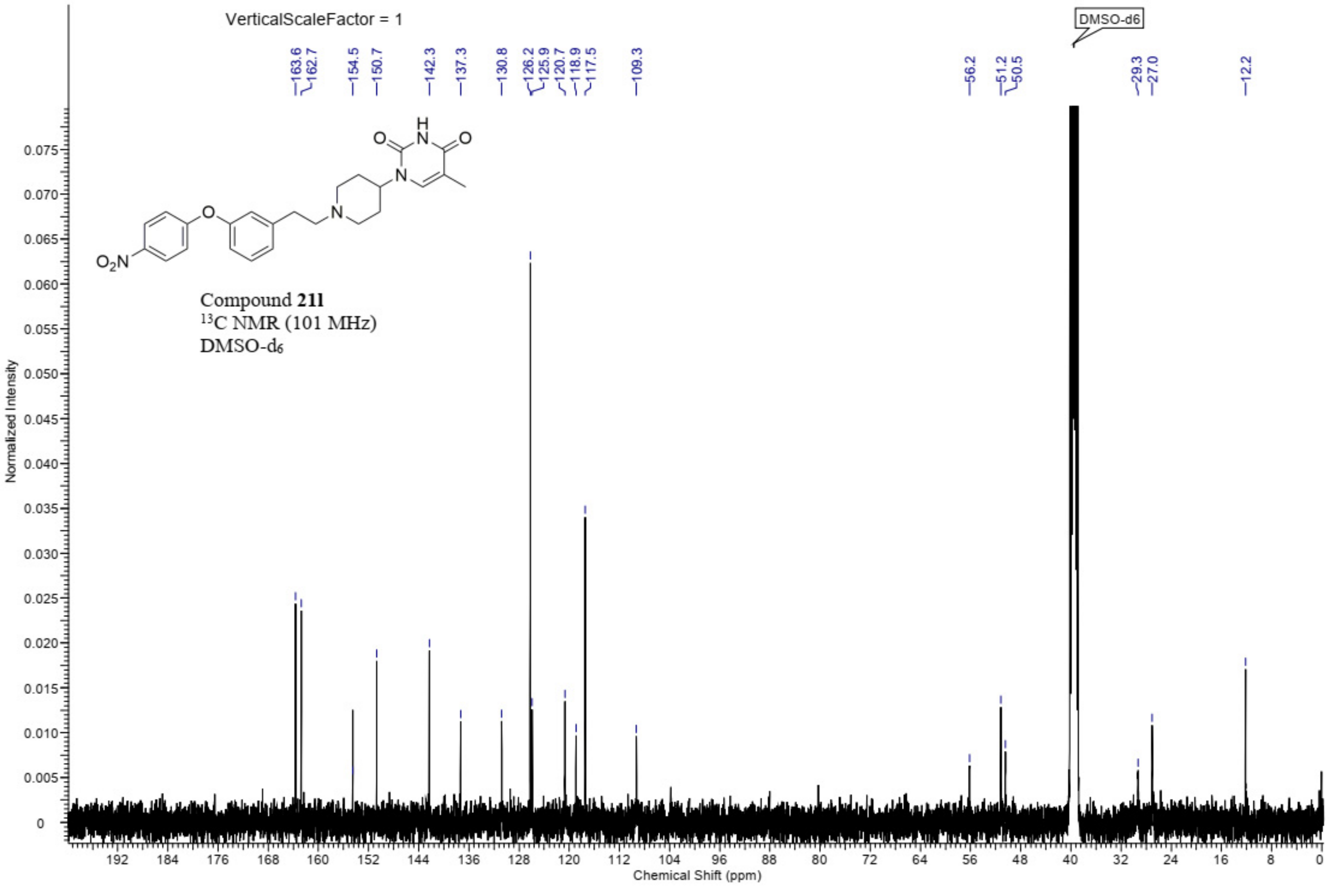

Figure S26. ^{13}C NMR spectrum of compound **21l**.

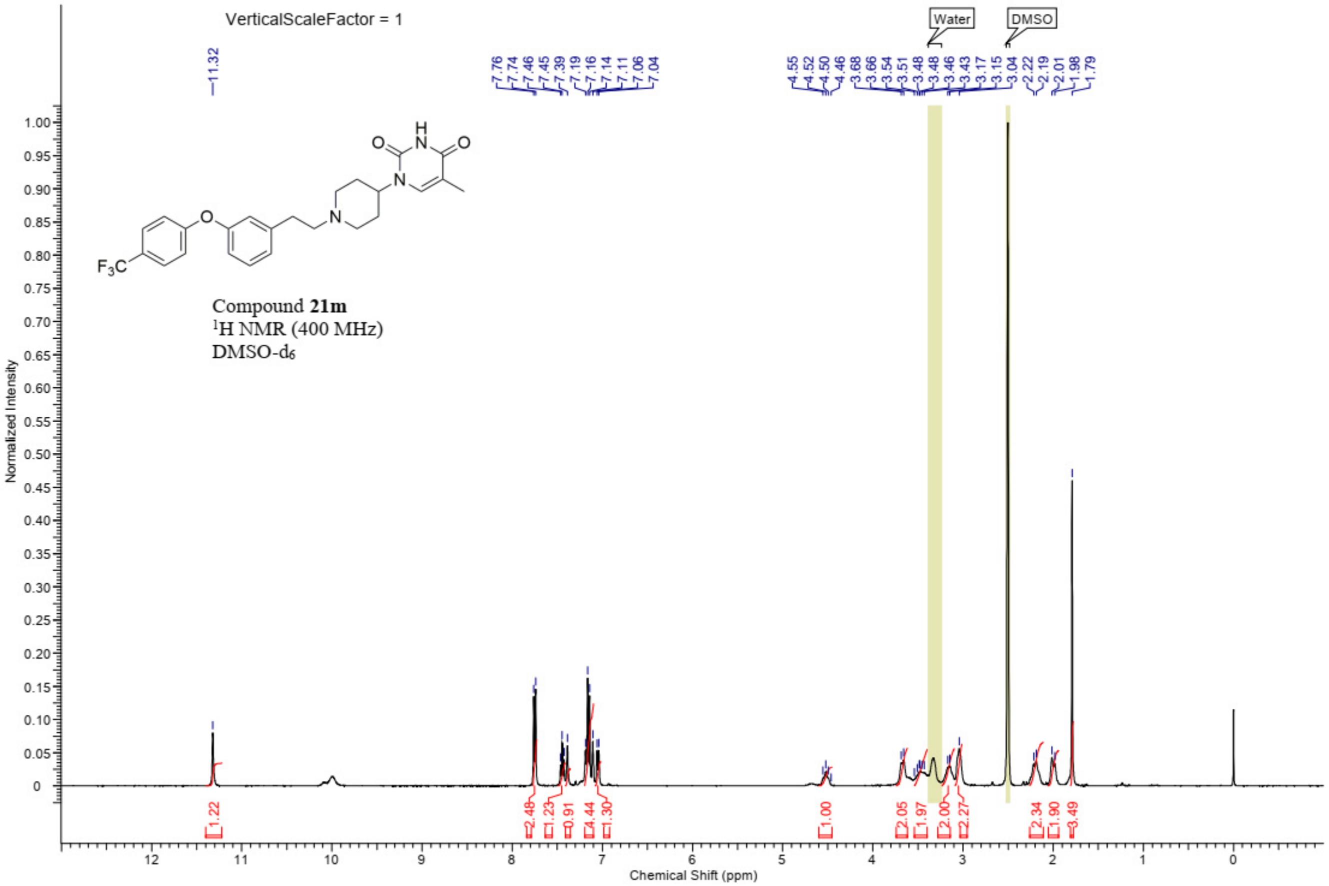

Figure S27. ^{1}H NMR spectrum of compound **21m**.

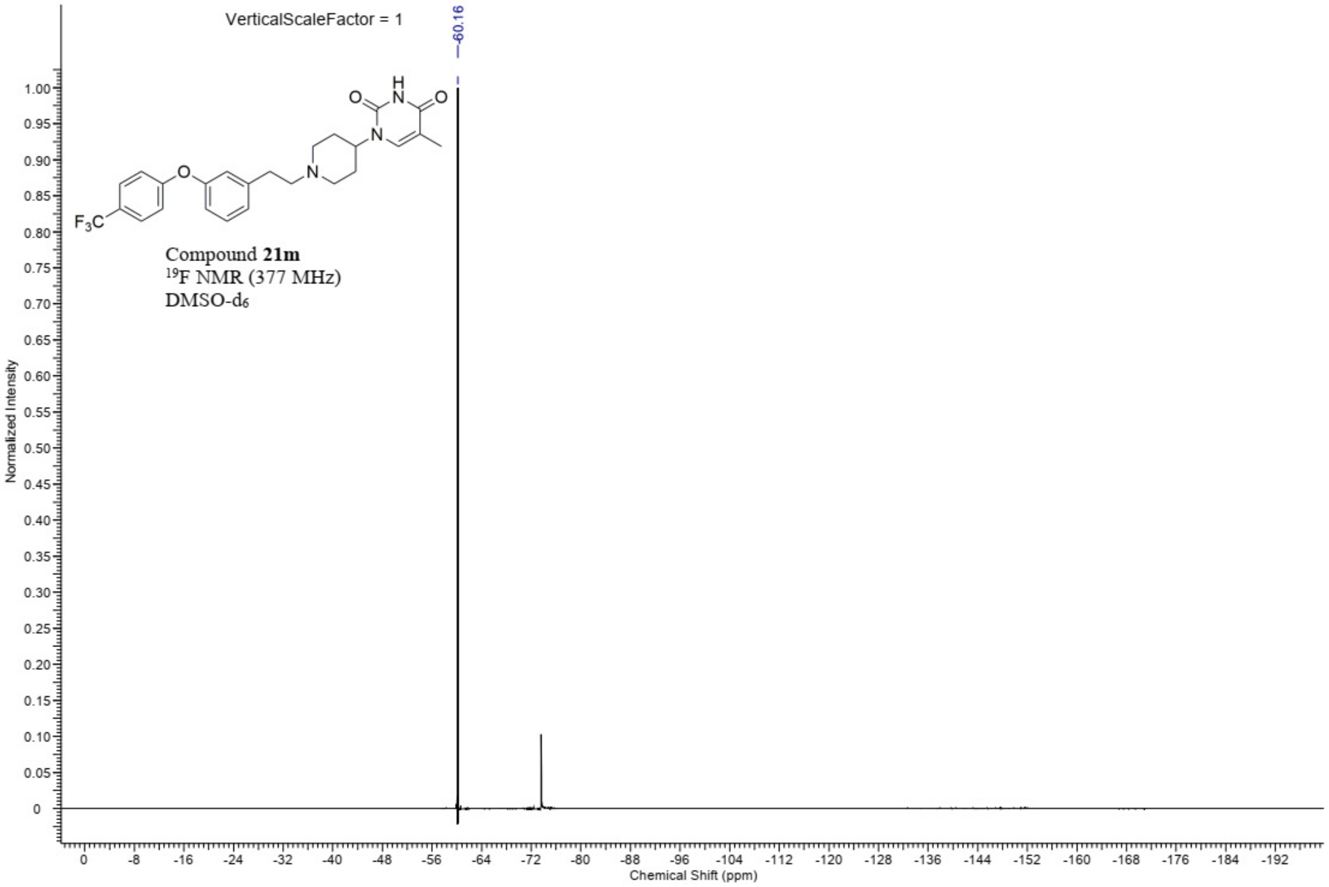

Figure S28. ^{19}F NMR spectrum of compound **21m**.

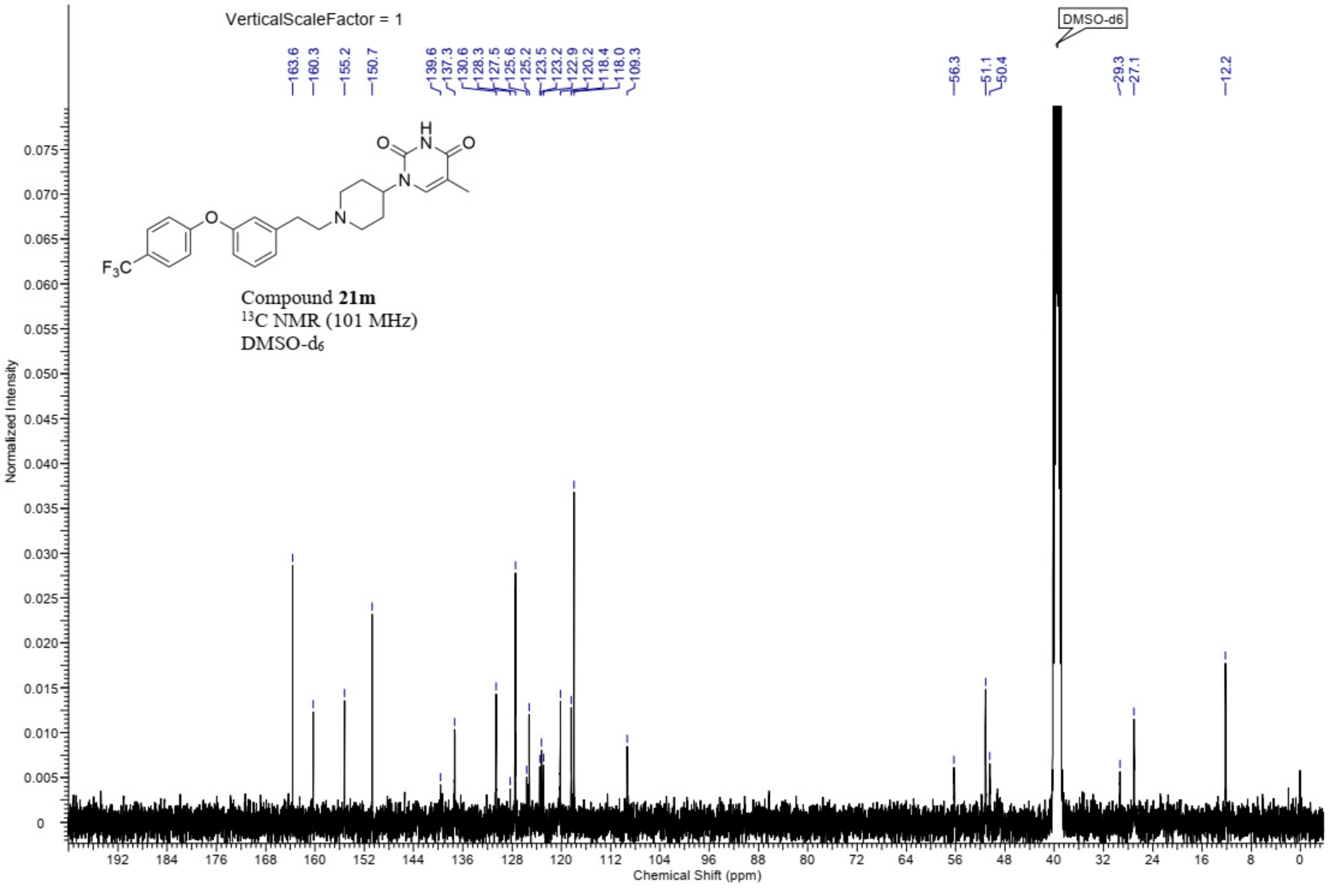

Figure S29. ^{13}C NMR spectrum of compound **21m**.

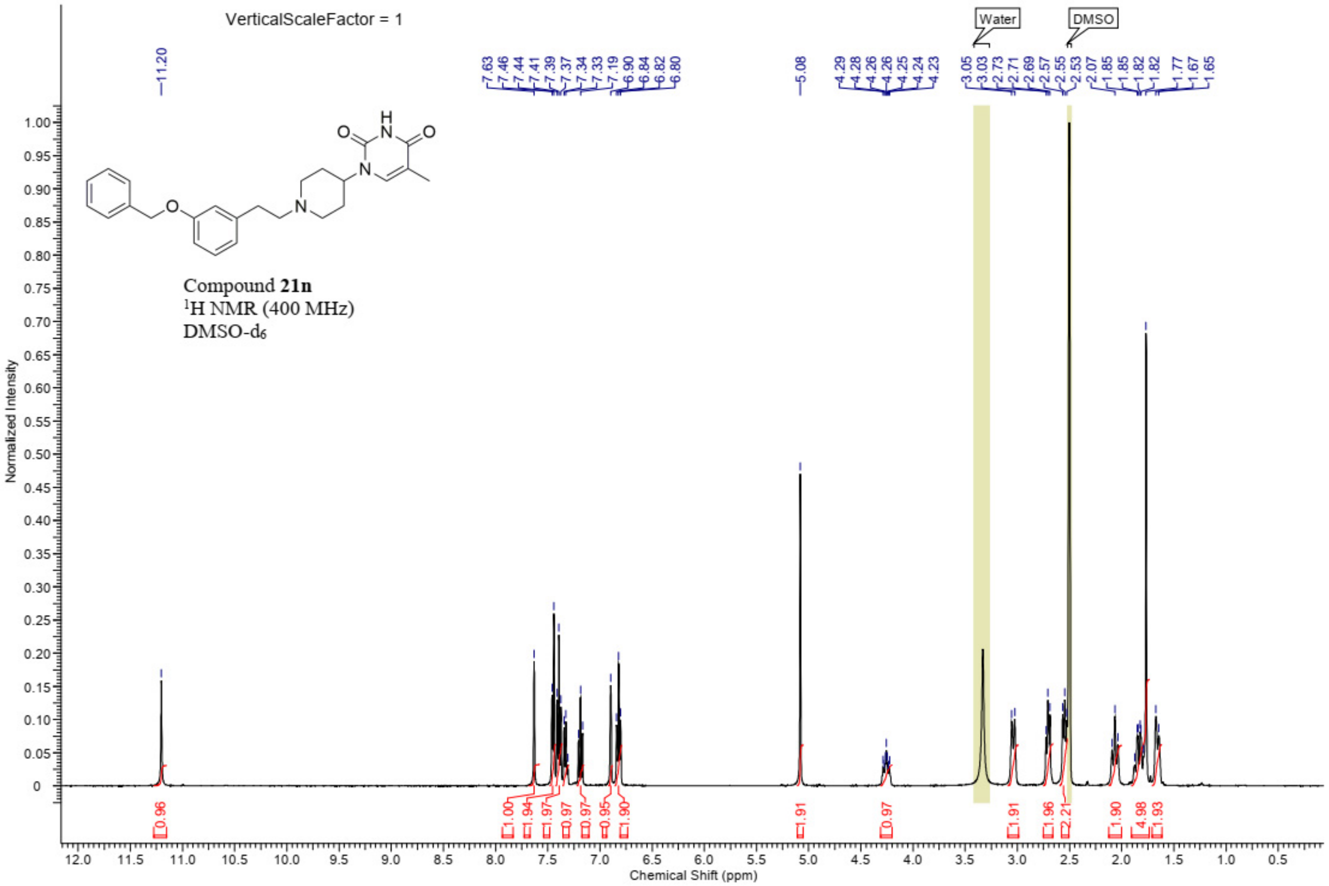

Figure S30. ^{1}H NMR spectrum of compound **21n**.

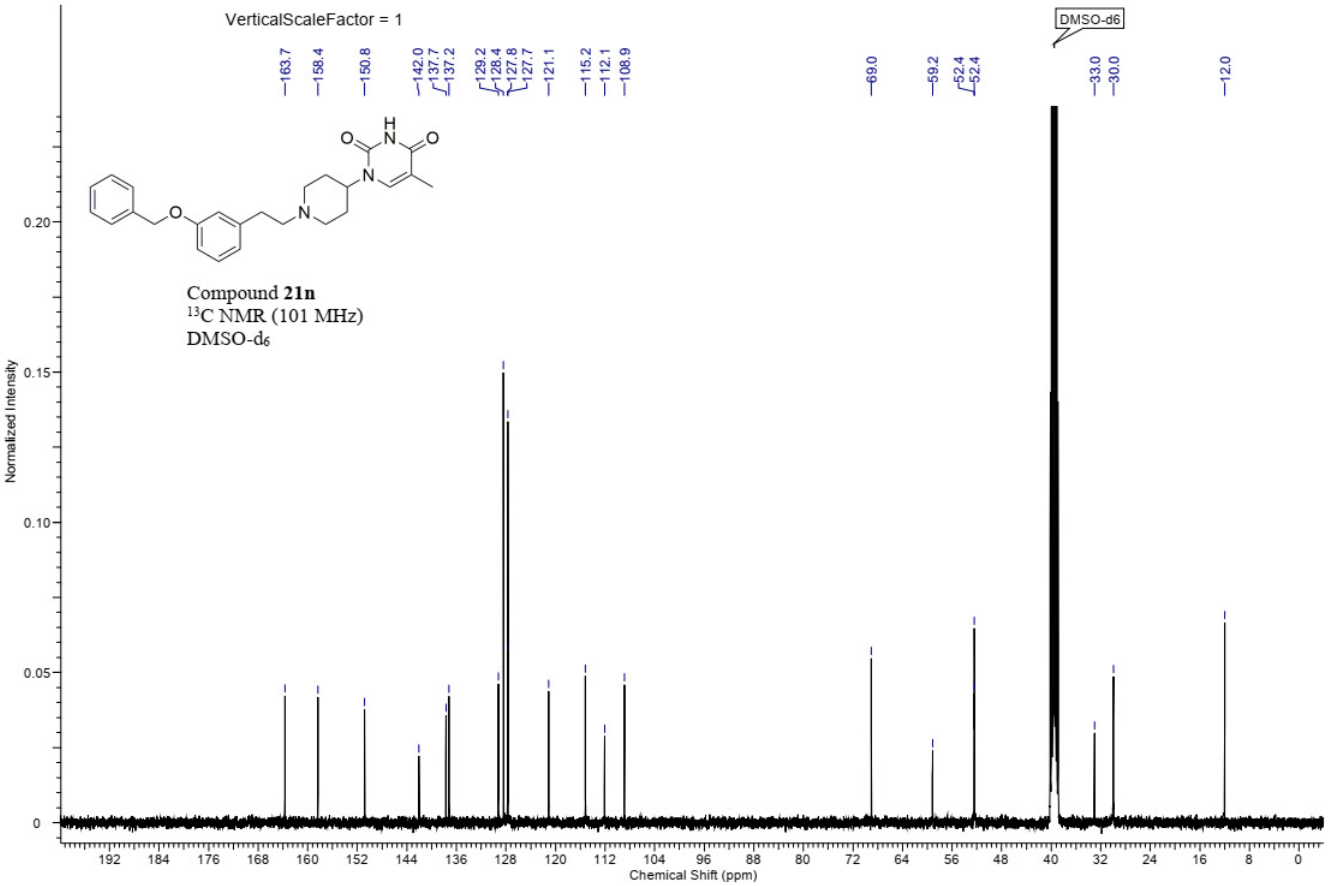

Figure S31. ^{13}C NMR spectrum of compound **21n**.

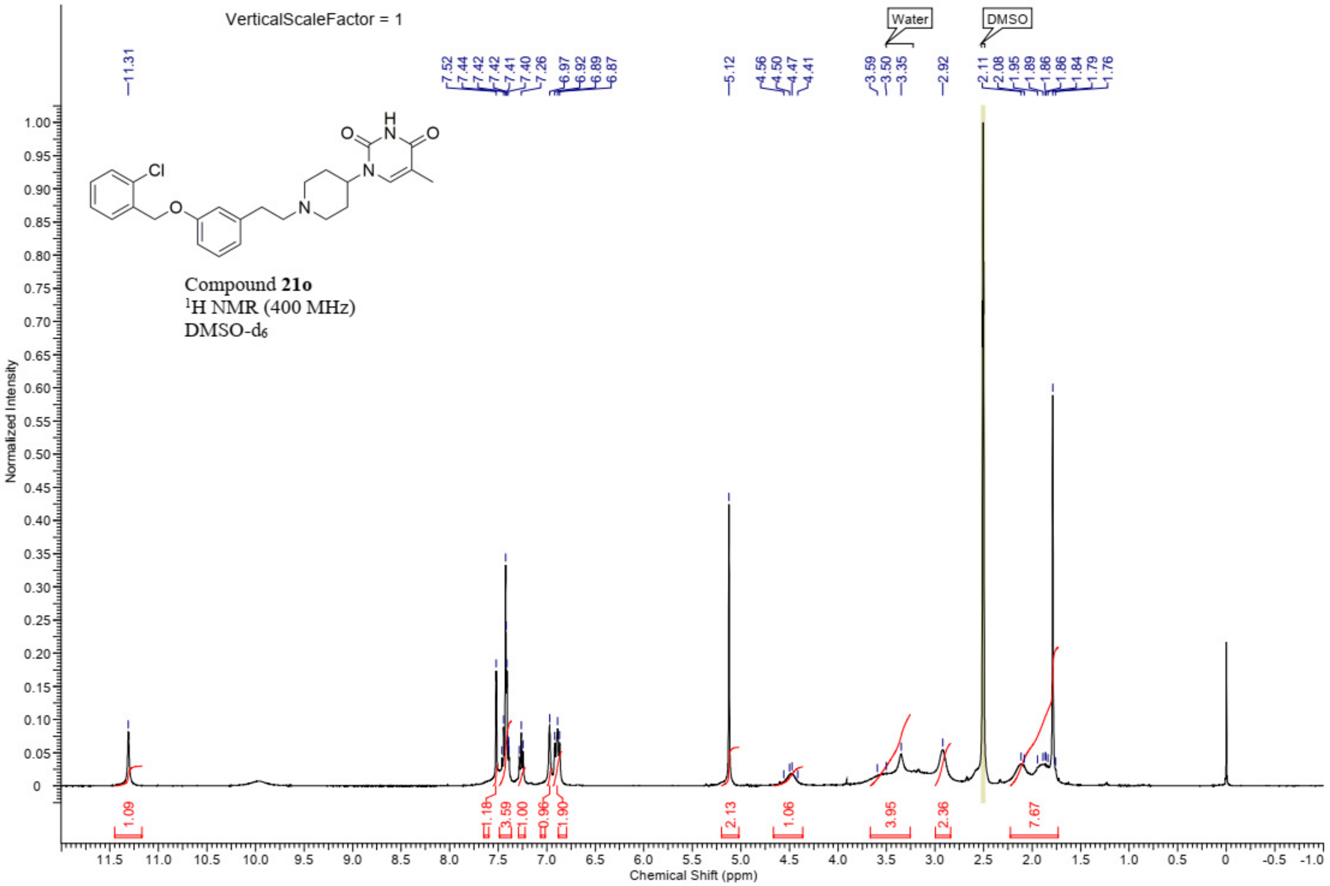

Figure S32. ^{1}H NMR spectrum of compound **21o**.

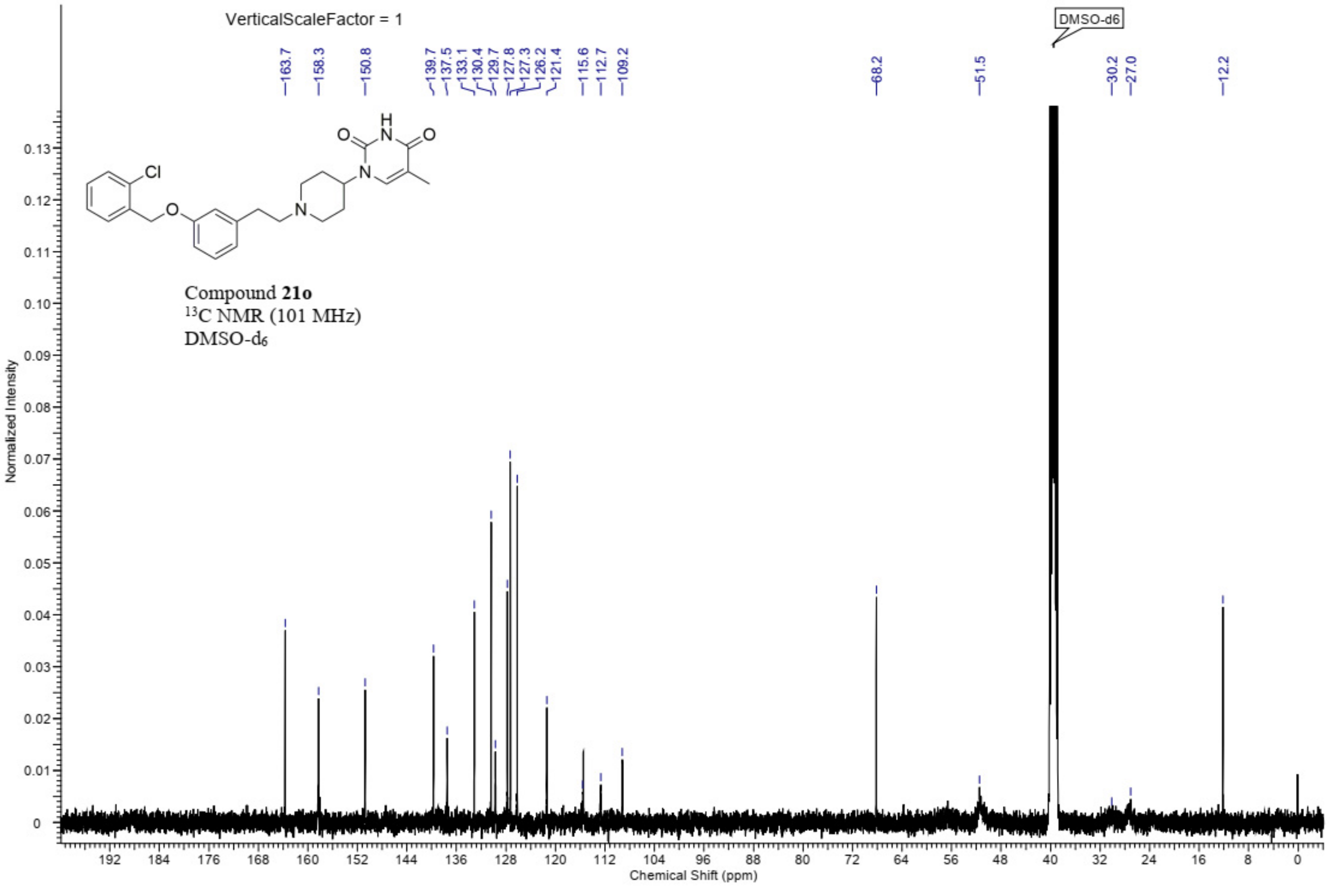

Figure S33. ^{13}C NMR spectrum of compound **21o**.

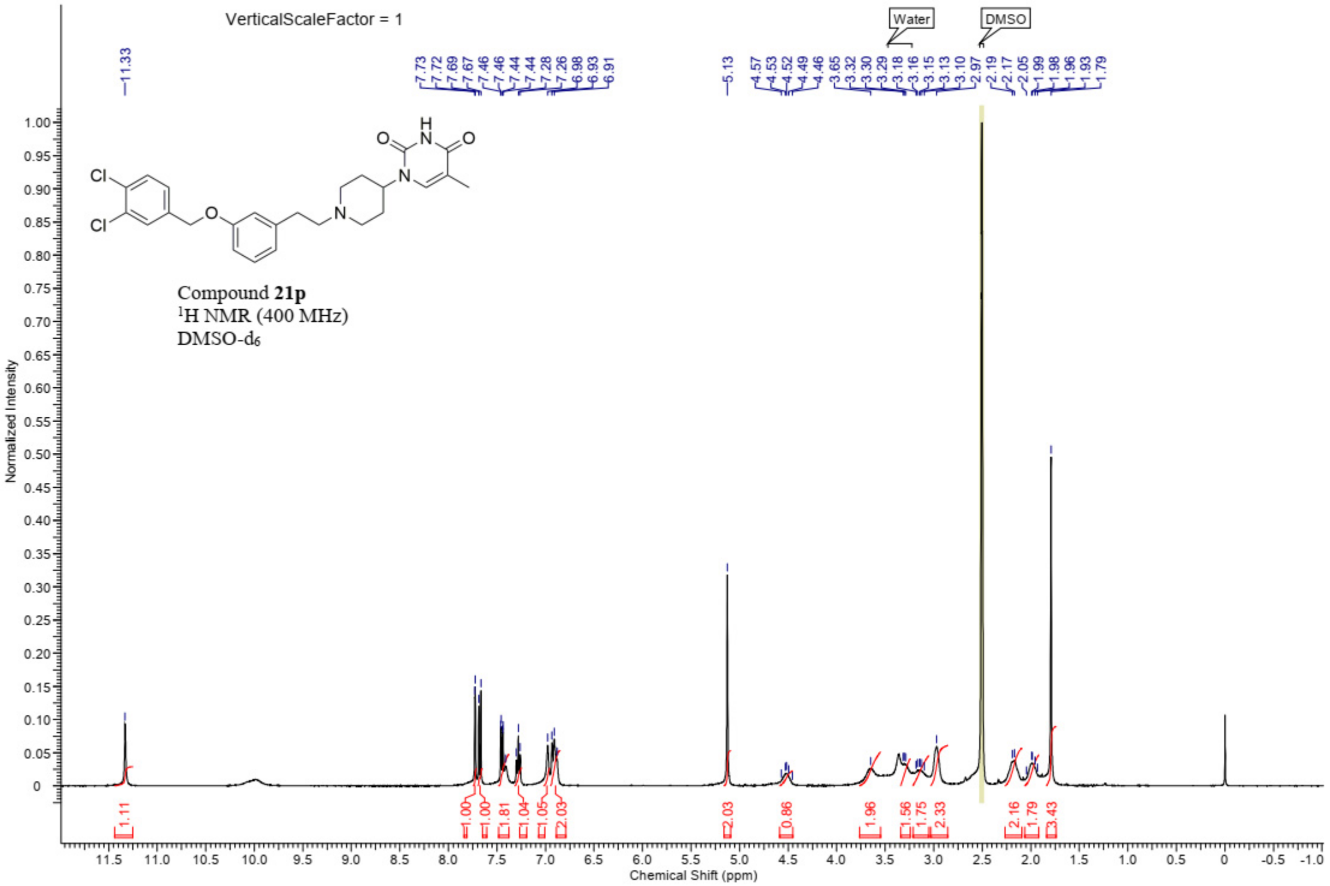

Figure S34. ^{1}H NMR spectrum of compound **21p**.

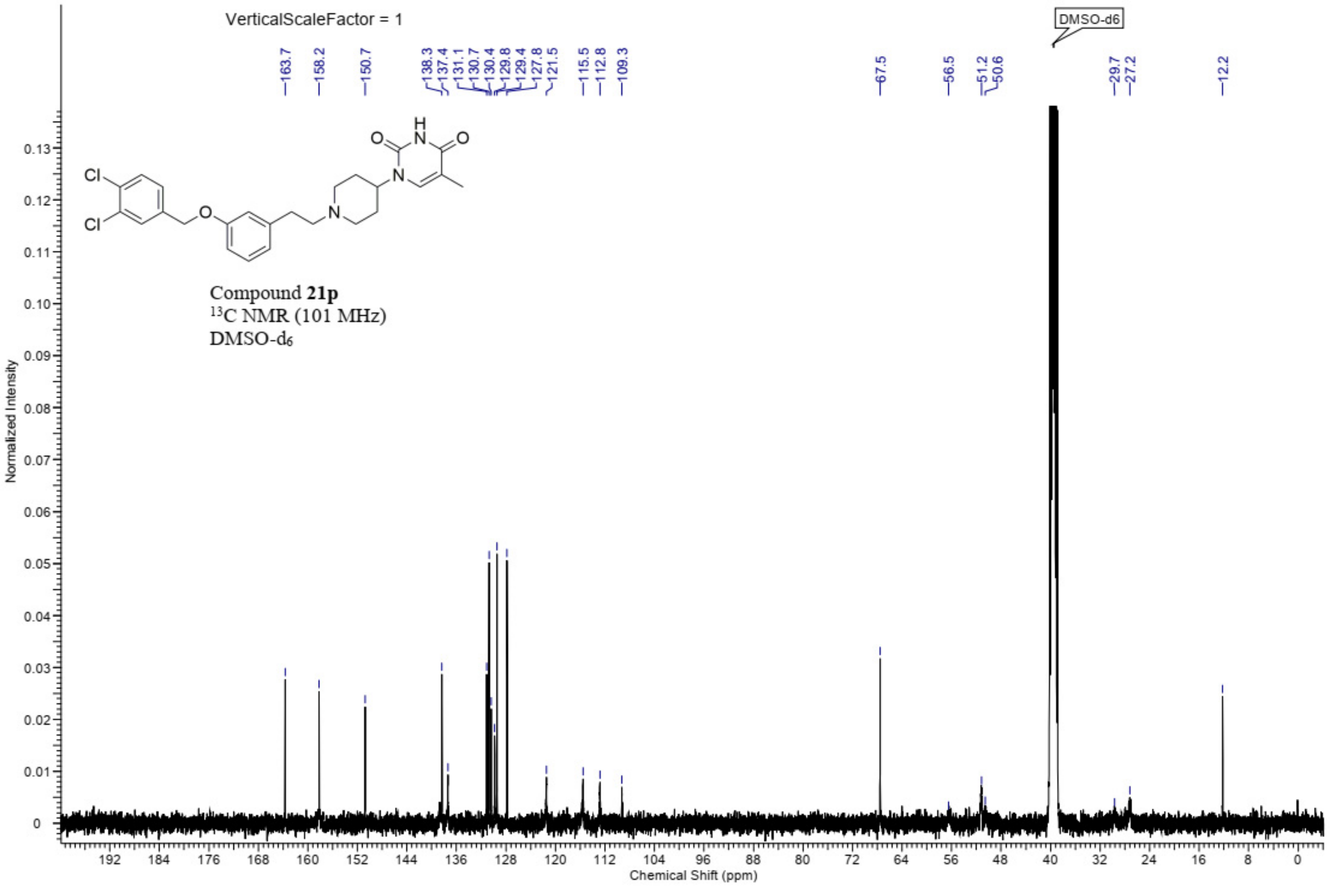

Figure S35. ^{13}C NMR spectrum of compound **21p**.

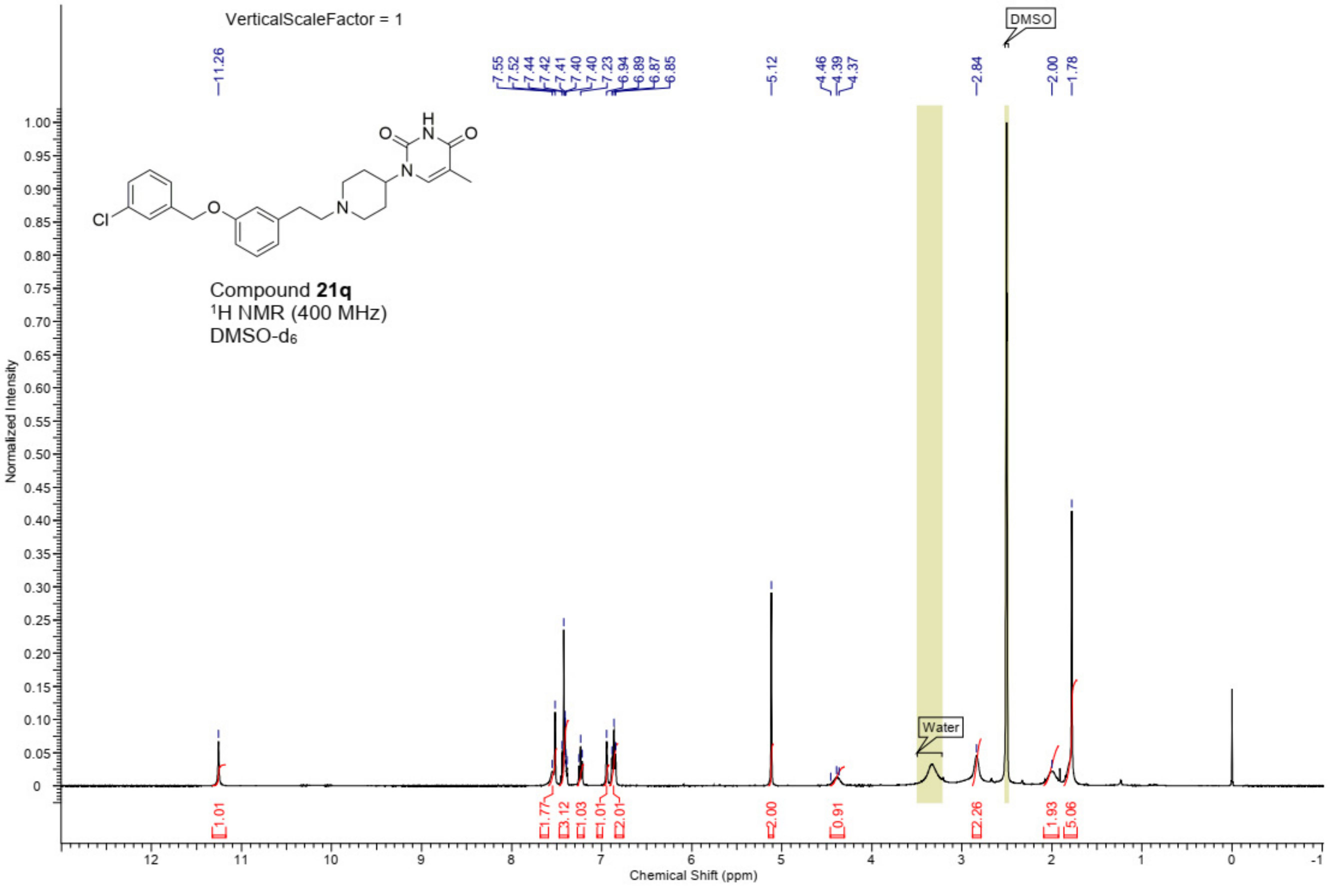

Figure S36. ^{1}H NMR spectrum of compound **21q**.

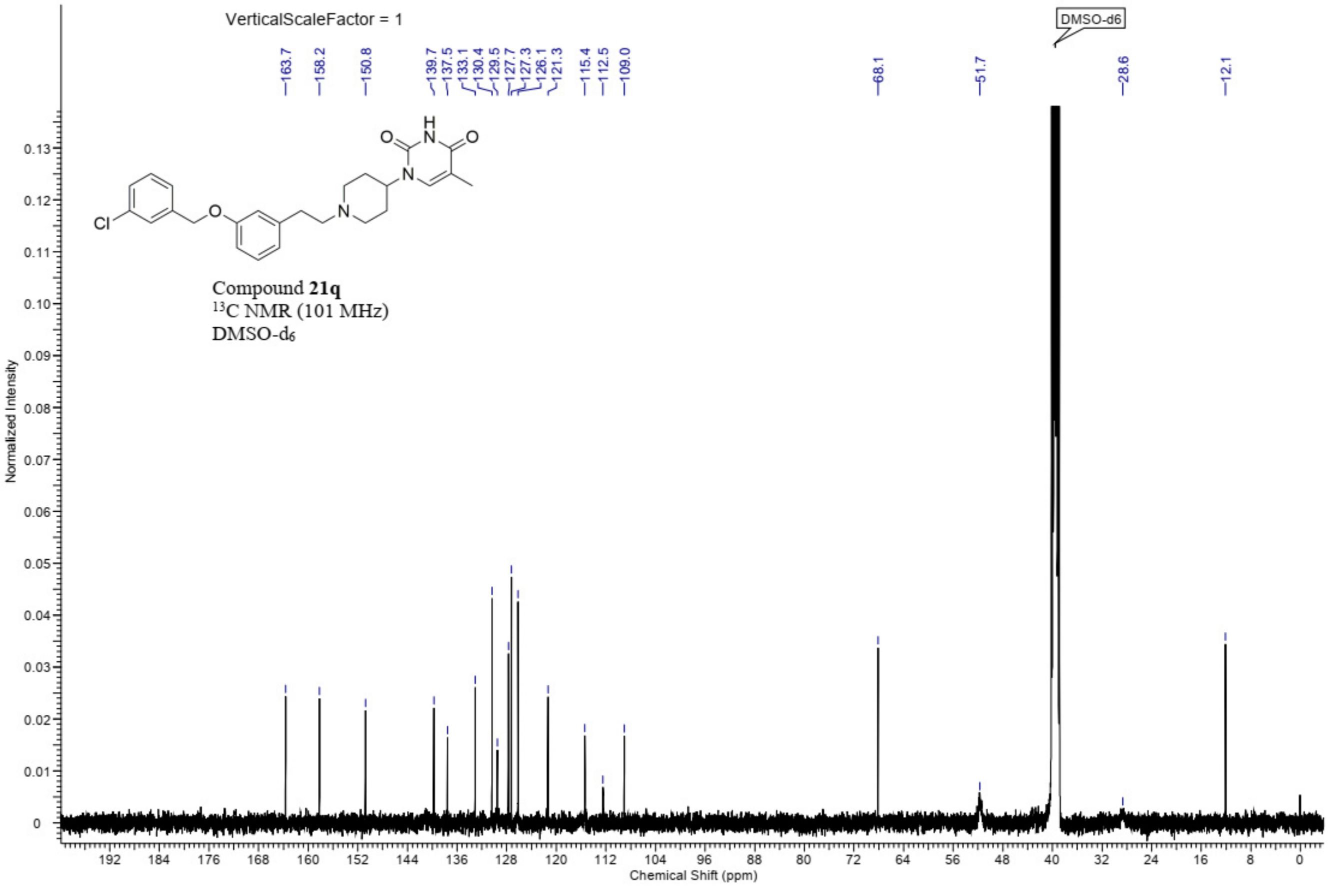

Figure S37. ^{13}C NMR spectrum of compound **21q**.

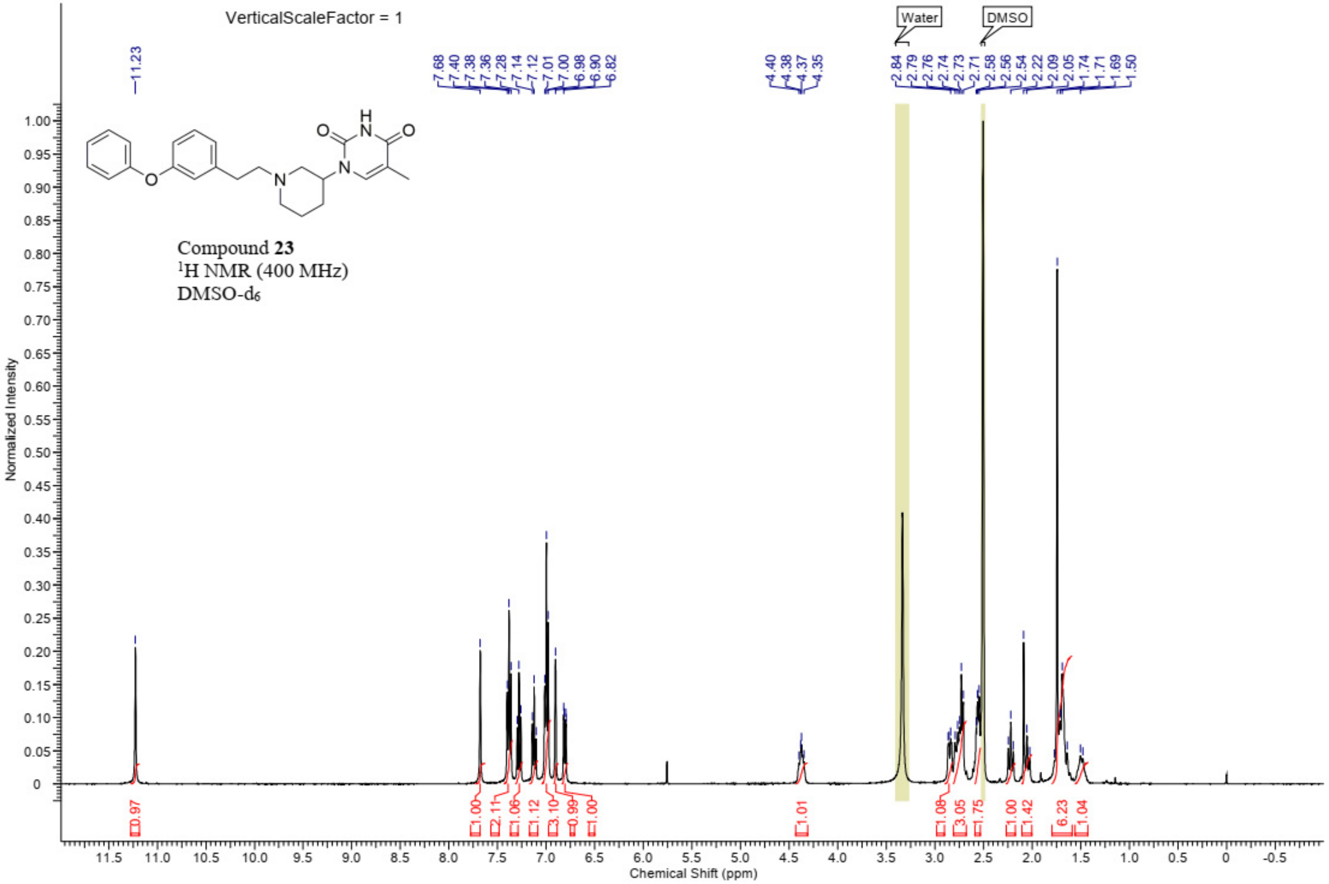

Figure S38. ^{1}H NMR spectrum of compound **23**.

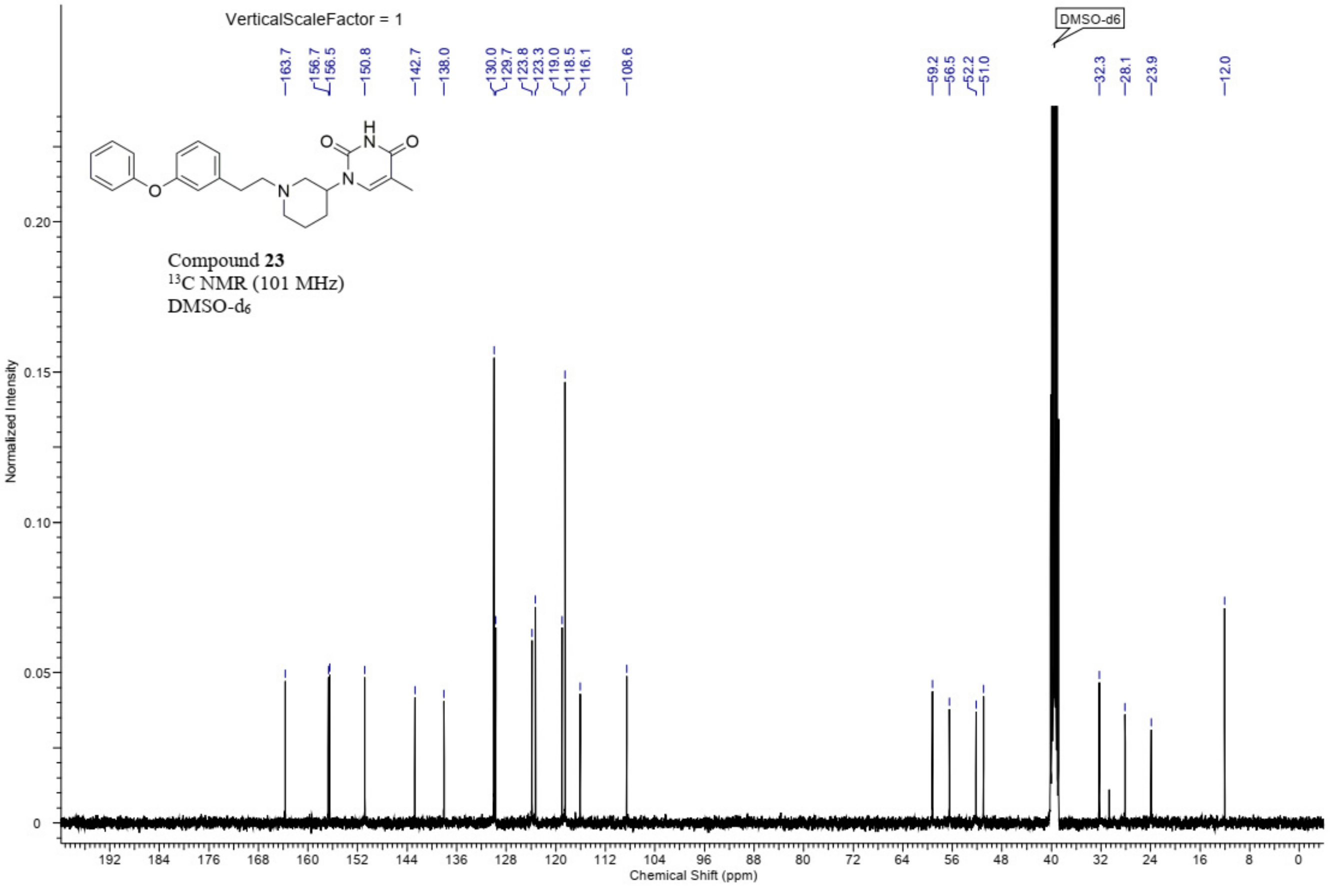

Figure S39. ^{13}C NMR spectrum of compound **23**.

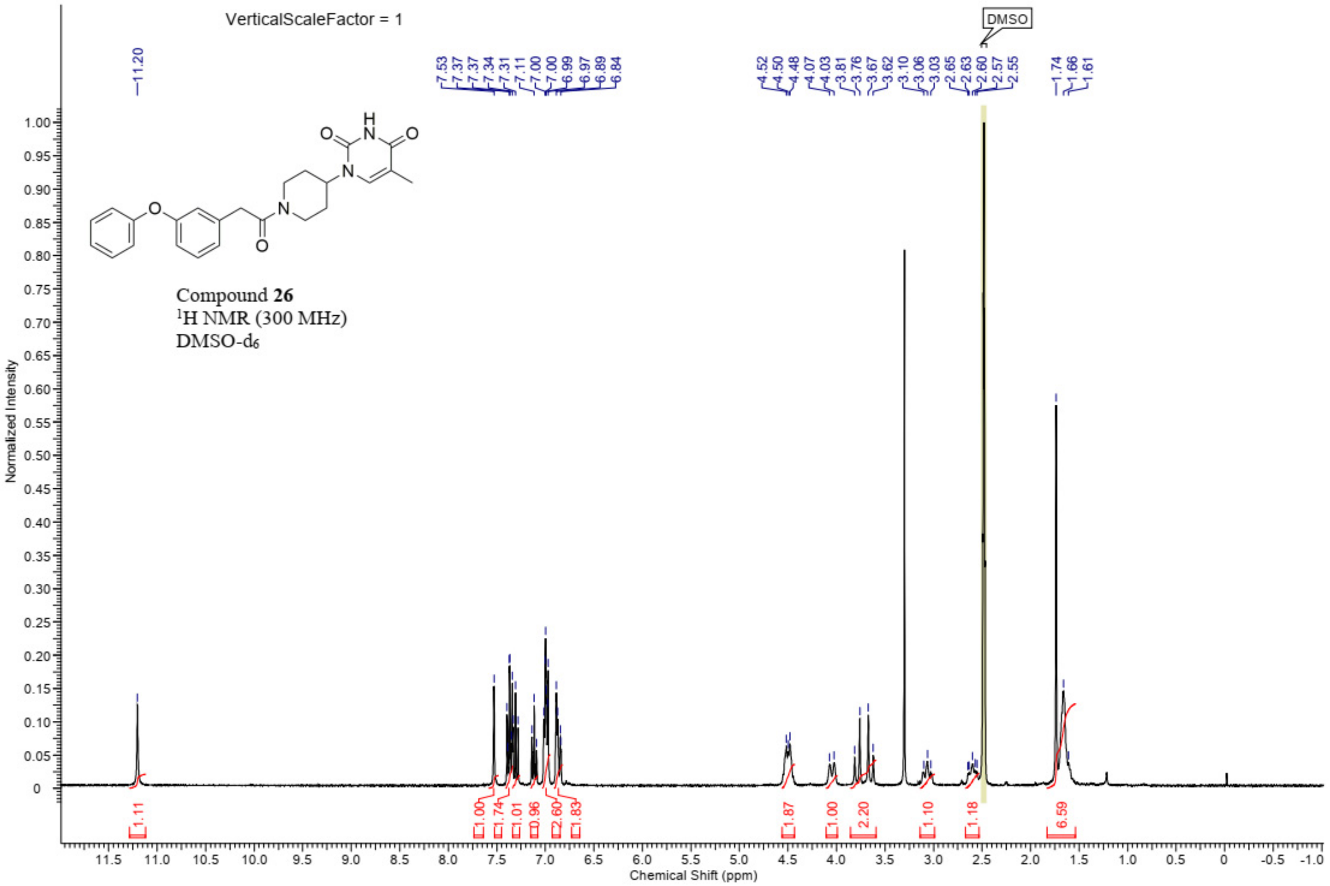

Figure S40. ^{1}H NMR spectrum of compound **26**.

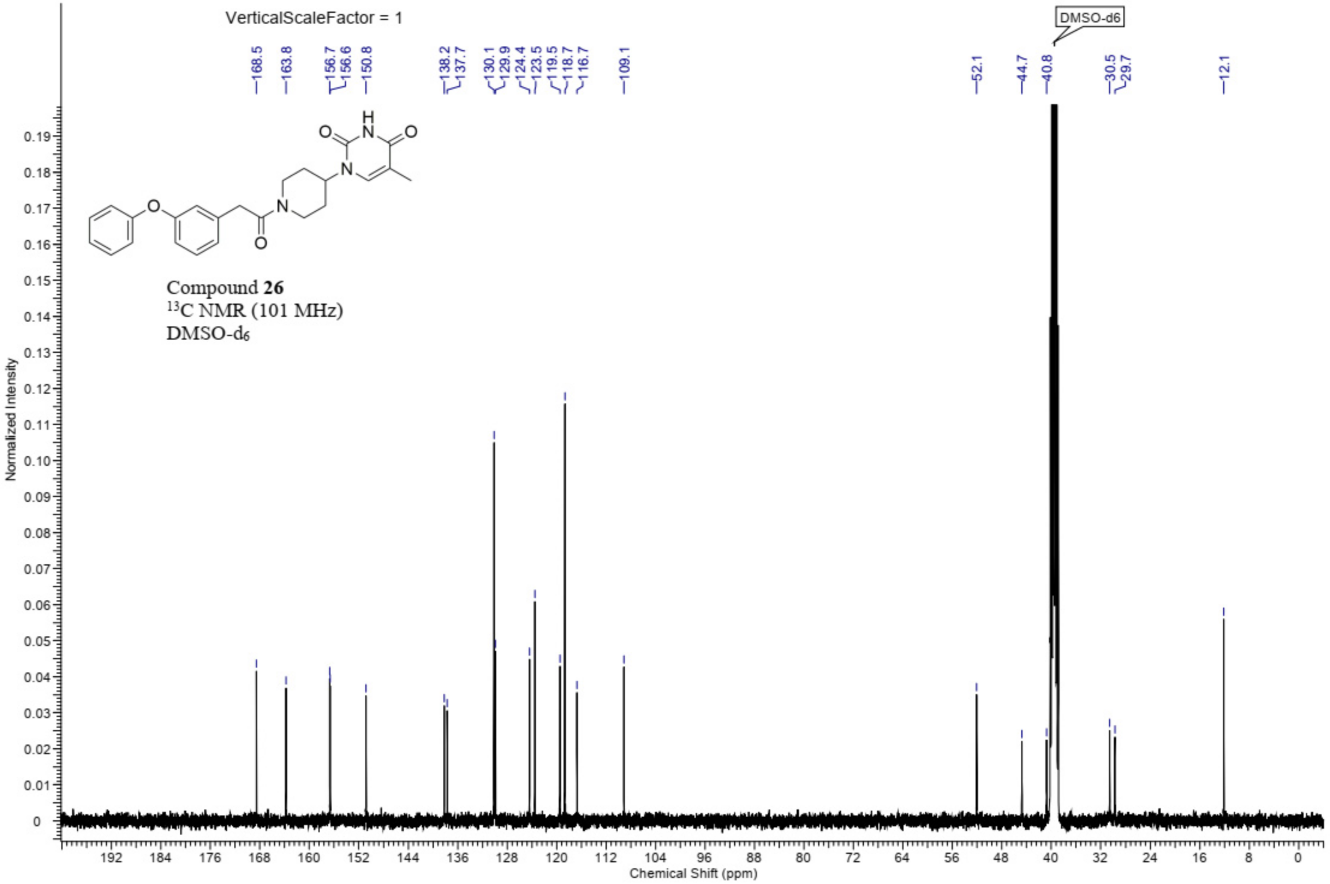

Figure S41. ^{13}C NMR spectrum of compound **26**.

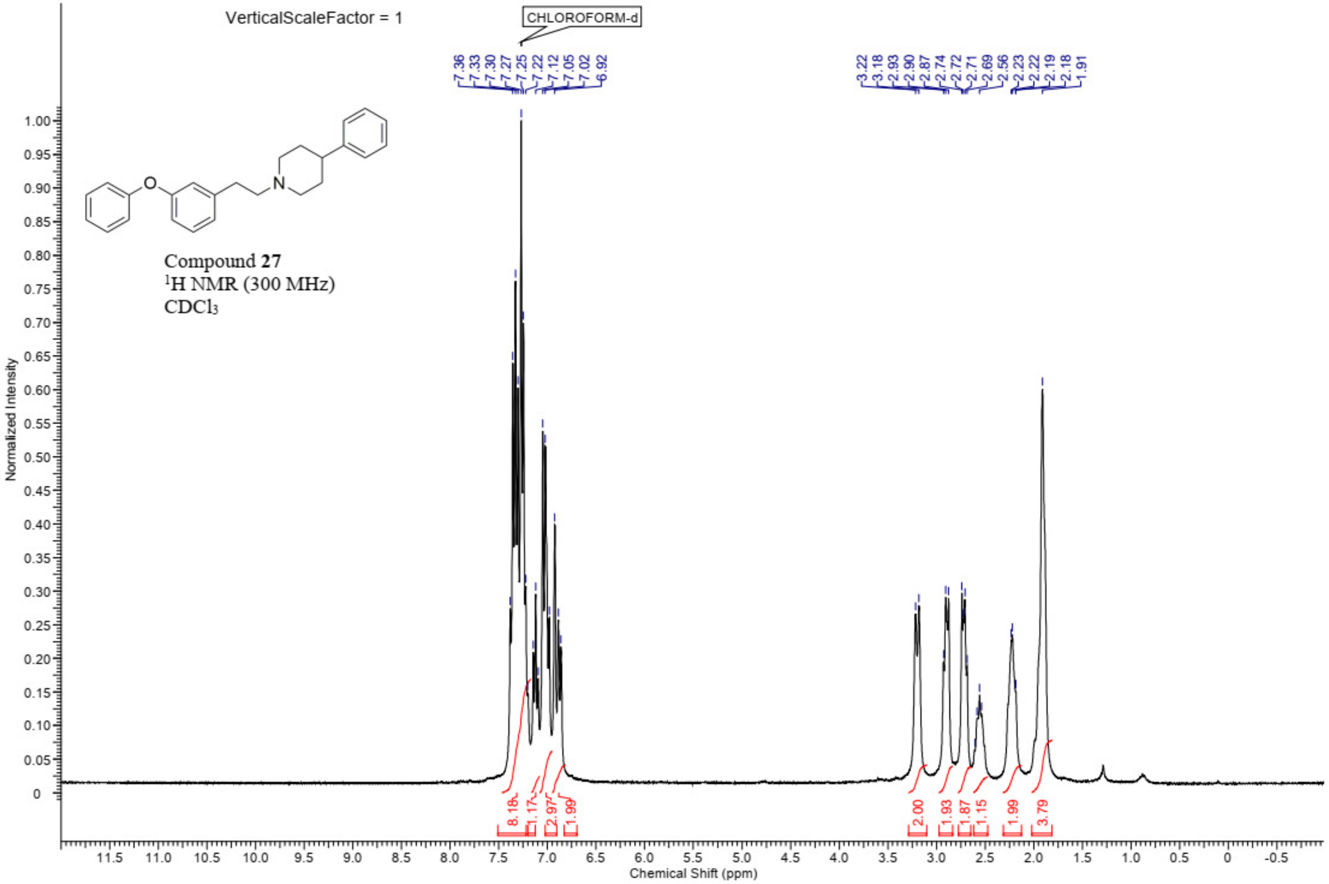

Figure S42. ^{1}H NMR spectrum of compound **27**.

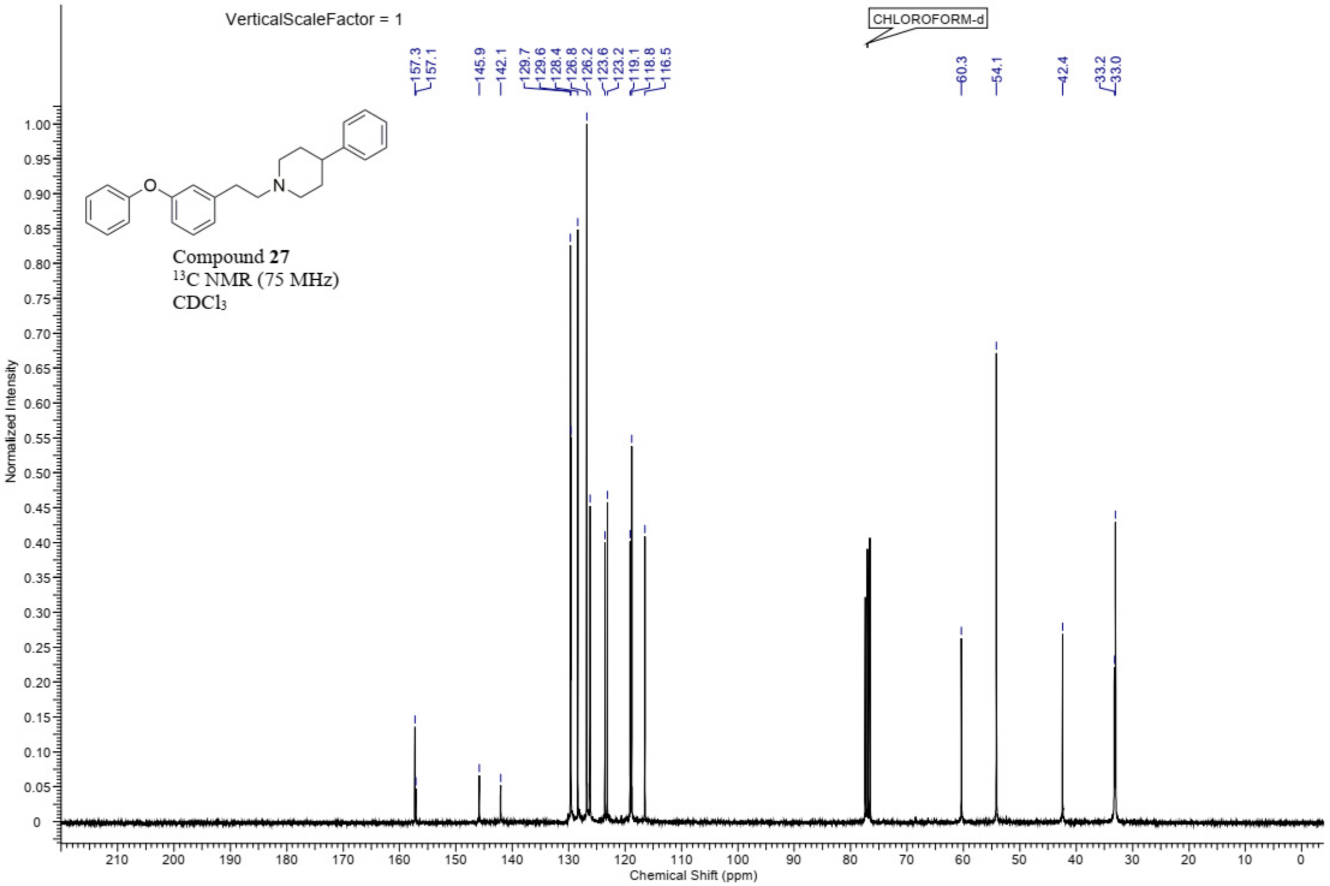

Figure S43. ^{13}C NMR spectrum of compound **27**.

© 2020 by the authors. Licensee MDPI, Basel, Switzerland. This article is an open access article distributed under the terms and conditions of the Creative Commons Attribution (CC BY) license (http://creativecommons.org/licenses/by/4.0/).
